# Antioxidants: Classification, Natural Sources, Activity/Capacity Measurements, and Usefulness for the Synthesis of Nanoparticles

**DOI:** 10.3390/ma14154135

**Published:** 2021-07-25

**Authors:** Jolanta Flieger, Wojciech Flieger, Jacek Baj, Ryszard Maciejewski

**Affiliations:** 1 Department of Analytical Chemistry, Medical University of Lublin, Chodźki 4A, 20-093 Lublin, Poland; 2 Chair and Department of Anatomy, Medical University of Lublin, Jaczewskiego 4, 20-090 Lublin, Poland; wwoj24@gmail.com (W.F.); jacek.baj@umlub.pl (J.B.); ryszard.maciejewski@umlub.pl (R.M.)

**Keywords:** antioxidants, natural extracts, antioxidant activity/capacity, green synthesis, nanoparticles

## Abstract

Natural extracts are the source of many antioxidant substances. They have proven useful not only as supplements preventing diseases caused by oxidative stress and food additives preventing oxidation but also as system components for the production of metallic nanoparticles by the so-called green synthesis. This is important given the drastically increased demand for nanomaterials in biomedical fields. The source of ecological technology for producing nanoparticles can be plants or microorganisms (yeast, algae, cyanobacteria, fungi, and bacteria). This review presents recently published research on the green synthesis of nanoparticles. The conditions of biosynthesis and possible mechanisms of nanoparticle formation with the participation of bacteria are presented. The potential of natural extracts for biogenic synthesis depends on the content of reducing substances. The assessment of the antioxidant activity of extracts as multicomponent mixtures is still a challenge for analytical chemistry. There is still no universal test for measuring total antioxidant capacity (TAC). There are many in vitro chemical tests that quantify the antioxidant scavenging activity of free radicals and their ability to chelate metals and that reduce free radical damage. This paper presents the classification of antioxidants and non-enzymatic methods of testing antioxidant capacity in vitro, with particular emphasis on methods based on nanoparticles. Examples of recent studies on the antioxidant activity of natural extracts obtained from different species such as plants, fungi, bacteria, algae, lichens, actinomycetes were collected, giving evaluation methods, reference antioxidants, and details on the preparation of extracts.

## 1. Introduction

Recently, much research has been devoted to free radical chemistry. There are undeniable pieces of evidence that free radicals are responsible for the oxidative damage of biomolecules such as proteins, lipids, or nucleic acids in the structures of cell nuclei and molecular membranes. Maintaining the balance between free radicals and antioxidants is a prerequisite for staying healthy. Thus, the control of oxidative stress processes may turn out to be fundamental in both the prevention and treatment of many diseases, such as diabetes, atherosclerosis, coronary artery disease, cancer, inflammation, liver diseases, cardiovascular diseases, cataracts, nephrotoxicity, and neurodegenerative processes accompanying aging. In order to maintain redox homeostasis, excess free radicals are neutralized by enzymes and non-enzymatic antioxidants, which, with the exception of a few produced by the human body, e.g., glutathione, uric acid, and uricinol, must be supplied with the diet. Since synthetic antioxidants butylated hydroanisole (BHA), butylated hydrotoluene (BHT), n-propyl gallate (PG) pose a potential health risk due to contamination with chemical precursors, toxic solvents, and the formation of hazardous by-products, natural antioxidants are an attractive alternative. For this reason, there is an extensive search for effective, non-toxic, and natural antioxidants. According to PubMed, in the last 5 years, over three thousand review articles that prove the effectiveness of natural antioxidants in preventing diseases caused by oxidative stress have been published. Therefore, antioxidants have become co-adjuvants utilized in conventional therapies with the aim of combating oxidative stress. Many natural antioxidants have been shown to have strong antiviral effects. The efficacy of flavonoids, i.e., (+)—catechin, luteolin, apigenin, quercetin, and quercetin 7-rhamnoside, has been proven in coronavirus infections (Porcine epidemic diarrhea virus (PEDV), Transmissible gastroenteritis virus (TGEV) [1,2,3]. In the absence of effective therapies for the treatment of diseases caused by coronaviruses, antioxidants may prove to be an effective alternative to fight the SARS- and MERS-CoV pandemic [4]. The site of action of antioxidants is the oxidative stress pathway, which plays a key role in coronavirus-induced pathogenesis. Diniz et al. [4] reviewed different effects of natural antioxidants against coronavirus covering reduction nucleocapsid (N) protein expression, inhibition 3C-like protease (3CLpro) [5,6,7,8] enzyme responsible for replication of SARS-CoV (quercetin and its derivatives), papain-like protease (PLpro) (isobavachalcone and psoralidin) [9], and helicase protein by affected ATPase activity (myricetin and scutellarein) [10]. A recently published review demonstrated the usefulness of antioxidants in the treatment of neurological disorders caused by COVID-19 [11]. However, reports that do not confirm the effectiveness of antioxidants in vivo cannot be ignored [12]. The activity of antioxidants is mainly limited by ADMET (Absorption, Distribution, Metabolism, Excretion, and Toxicology)processes related to poor absorption caused by restrictions in the penetration of cell membranes and degradation that occurs in the stomach and intestines. It has also been reported that low molecular weight antioxidants lose their ability to scavenge free radicals inside cells. This is especially true for the scavenging of the hydroxyl radical (OH^•^), superoxide (O_2_^•−^), and H_2_O_2_ [13,14].

The sources of natural antioxidants are mainly plants, i.e., edible vegetables, fruits, spices, and herbs, which are rich in vitamins, phenolic compounds, carotenoids, and microelements [15,16,17]. However, it should be emphasized that the antioxidant activity is different for different varieties and morphological parts of natural resources. In addition, the activity of natural products is influenced by many other factors, such as climatic and soil conditions or harvest time. They hinder the standardization of natural products to a large extent. Due to the fact that natural antioxidants have the ability to inhibit the processes of oxidation and the growth of microorganisms, including many pathogenic ones, e.g., *Salmonella* spp. and *Escherichia coli* [18], they are more and more often used as preservatives in food products [19] or as packaging ingredients for food [20]. In recent years, a large body of evidence has been published that natural antioxidants increase the stability of edible oils [21,22,23], the stability of carotenoid dyes, and the aroma of fruit juices [24] and that they work well as additives in meat products [25,26] and even in bakery products [27], successfully replacing artificial preservatives and stabilizers. It should be emphasized that the choice of bioactive compounds for the food industry is significantly limited due to the obvious taste requirements [28] and the need for approval by EFSA (European Food Safety Authority) or FDA (American Food and Drug Administration). Currently, we can observe an interesting trend in the strategy of using by-products of the processing industry [29,30]. This is related not only to environmental protection or economic reasons but to the fact that they have a significant content of bioactive substances, exceeding that in the flesh of fruit, such as polyphenols in apple and olive pomace [31], lycopene in tomato pomace [32], phenolic compounds from the group of flavonoids (anthocyanins, catechins), and phenolic acids and stilbenes in grape skins [33] or citrus fruits [34].

In recent years, natural extracts have become attractive, also due to the rapid development of nanotechnology. As a source of substances with reducing potential, they have replaced the toxic reagents used in chemical synthesis and ushered in the era of the so-called biogenic synthesis and nanobiotechnology [35].

Numerous spectroscopic, biochemical, and electrochemical assays are used to test antioxidant abilities, which are still modified so that they can effectively assess the potential of antioxidants, taking into account the variability of their mechanisms of action. They are usually based on a free radical scavenging reaction or the prevention of their formation by the addition of an antioxidant. Various techniques used for this purpose differ in terms of repeatability and costs associated with the necessity of using specialized equipment. Valuable reviews, published in recent years, describe problems related to (i) the mechanisms responsible for the antioxidant activity [17]; (ii) the antioxidant activity of natural extracts prepared from various plant species [36], microorganisms [37], and food ingredients [38]; and (iii) the preventive role of antioxidants in various diseases such as diabetes [39], human gut diseases [40], and cancer [41], as well as the use of natural extracts for the synthesis of nanoparticles [42].

This review gathers together issues related to antioxidants (classification, natural sources, measurement of antioxidant activity) as well as their application in nanotechnology. Within the review, two main issues can be distinguished:

(i) The wide range of industrial and biomedical applications of antioxidants requires effective and rapid in vitro tests to evaluate total antioxidant activity. Various methods were collected in the review, i.e., chromatographic, spectrometric, and electrochemical. Particular attention was paid to the method based on metallic nanoparticles, which are used as optical probes (SNPAC). The method is useful for measuring the antioxidant activity of both simple chemical compounds and mixtures of natural origin. The SNAPC tests are effective in assessing electron transfer but are not used very often. The review includes information on the extracts from plants, lichens, fungi, algae, and actinomycetes (reference antioxidants, extraction process, antioxidant activity tests, and activity parameters).
(ii) Natural extracts as a source of both reducing and stabilizing substances are used for the green synthesis of nanoparticles. The review includes examples of the synthesis of metallic/metal oxides of nanoparticles using extracts from various plant species and microorganisms (yeast, algae, cyanobacteria, fungi, and bacteria). The information collected allows us to trace the links between the type of antioxidant, its origin, activity, and suitability for the efficient synthesis of nanoparticles. Extensive data were collected on the methods of extract preparation, antioxidant activity tests, detection methods, NPs synthesis conditions, and the morphology of the obtained nanoparticles.

This review highlights recent trends in antioxidant research, measurement of antioxidant activity, biogenic nanoparticle synthesis, and nano-drug delivery systems.

## 2. Free Radicals/Antioxidants

### 2.1. Free Radicals vs. Oxidative Stress

Free radicals can be defined as highly reactive species that contain an unpaired electron in the valence shell. They can donate this electron but also accept it from other molecules, acting as an oxidant or reducing agent [43]. In the human body, reactive forms (RS) come from metabolic processes involved in the respiratory chain, phagocytosis, prostaglandin synthesis, and the cytochrome P-450 system [44]. 

The most reactive species found in biological systems include the hydroxyl radical (OH^●^), which is formed by attaching three electrons to an oxygen molecule, e.g., as a result of the Fenton reaction, and the superoxide radical (O_2_^●−^), which is formed mainly in mitochondria, as a byproduct of electron transport in the respiratory chain. Other reactive forms of oxygen (ROS), nitrogen (RNS), and chlorine occurring as free radicals and nonradicals that as oxidizing agents can be easily converted into radicals are listed in Table 1 [45,46].

ROS/RNS generated in oxygen metabolism are necessary in the regulation of gene expression, cell proliferation, apoptosis, the processes of protein phosphorylation or calcium concentration in cells, activation of proteins controlling cell division, and elimination of microorganisms. Free radicals are also generated under the influence of external sources, such as exposure to X-rays, ozone, smoking, air pollution, and industrial chemicals [48,49]. There is a balance in the cell between RS production and its neutralization by defense systems. Under physiological conditions, this balance is slightly shifted in favor of pro-oxidative conditions, providing continuous, mild oxidative stress [50].

Each disturbance of this particular balance may lead to the development of oxidative stress, i.e., a state in which the oxidizing potential increases to a level that threatens the stability of cellular structures [51]. Under oxidative stress, biologically important macromolecules such as DNA, proteins, carbohydrates, and lipids are damaged. The excess of free radicals changes their structure and thus the physiological functioning of the cell by disrupting redox signaling and the accumulation of cytotoxic compounds, such as malonyl dialdehyde or 4-hydroxynonenal [52,53].

There is evidence that free radicals can accumulate throughout the body with age, initiating the aging process, as well as various neurodegenerative diseases such as Alzheimer’s disease, Parkinson’s disease, muscular dystrophy, and atherosclerosis [54]. An imbalance between ROS and the antioxidant defense system has also been recognized in the induction of diabetes and age-related eye disease [55]. Currently, it is believed that oxidative stress has a significant negative impact also on inflammatory diseases, cancer, ischemic diseases, immunodeficiency syndrome, hypertension, alcoholism, smoking-related diseases, and many others [56,57,58,59,60,61]. Oxidative stress was first described and defined by Sies in 1991 [62].

The reasons for the occurrence of oxidative stress may be (i) an increase in the rate of ROS production, (ii) deficiencies of low-molecular-weight antioxidants, and (iii) inactivation of enzymes with antioxidant activity. Increased and/or prolonged state of oxidative stress may cause serious damage to the cell and even lead to its death [63]. Therefore, the current discussions focus on the role of free radicals in the pathogenesis of many diseases and the usefulness of antioxidants in their potential therapy [55,64,65].

Antioxidants are produced by the protective system of various organisms in order to respond to the destructive effects of free radicals. Antioxidants are able to reduce the damage caused by ROS/RNS and even chlorine. The action of the protective system may limit the negative effects of free radicals by preventing the formation of reactive radicals or by interrupting free radical reactions [66].

### 2.2. Antioxidants

**Figure 1:**
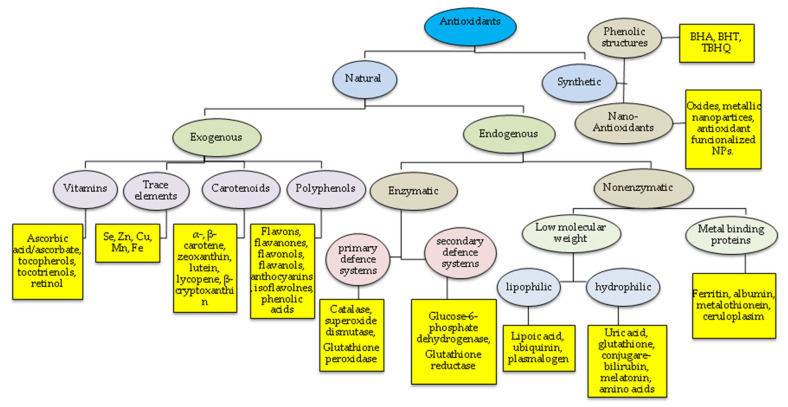
Antioxidants classification.

Antioxidants act by delaying or preventing the oxidation of other chemicals. The first studies on the role of antioxidants in biology focused on their use in preventing unsaturated fats from going rancid [67,68,69]. However, the milestone that led to the understanding of the role of antioxidants for living organisms was the identification of vitamins A, C, and E [70] and the understanding of the mechanism of lipid peroxidation prevention by vitamin E [71]. The classification of antioxidants, along with the most representative examples, is shown in the diagram (Figure 1). Antioxidants are usually classified into enzymatic and non-enzymatic. Among them, there are various compounds with different modes and places of action and different final effects. This diversity determines the individual role of each of them in the body. It should be emphasized that the network of interacting antioxidant enzymes, such as superoxide dismutase enzymes (SODs), catalase (CAT), glutathione peroxidase (GPx), and glutathione reductase (GRd), shows the highest antioxidant defense effectiveness [72].

Low-molecular-weight antioxidants, including vitamin C, E, coenzyme Q, carotenes, glutathione, and trace elements, are also responsible for inactivating reactive radicals. Some of them, including glutathione, ubiquinone, albumin and metallothioneins, and uric acid, are produced in the body [73], but most are exogenous compounds derived from natural sources such as plants (flavonoids, phenolic acids, carotenoids, stilbenes, coumarins, lignans, organosulfur compounds, vitamins) or minerals (selenium, zinc, manganese) provided with the diet. When endogenous antioxidants involved in free radical defenses cannot protect the body against ROS, there is a need for exogenous antioxidants. Almost all living organisms, both prokaryotes and eukaryotes, are capable of producing bioactive compounds.

Many of the naturally occurring antioxidants are now isolated, fully characterized, and available for various applications as prophylactic and therapeutic agents to inhibit the adverse effects generated by ROS [74,75].

A good diet that includes fruit, tea, wine, vegetables, and grains is a rich source of antioxidants. Some drugs, apart from their therapeutic effect, also have antioxidant effects, e.g., captopril belonging to angiotensin-converting enzyme (ACE) inhibitors, N-acetylcysteine [76], or dihydropyridine calcium antagonists [77]. However, the concentrations used in the therapy do not provide antioxidant activity in vivo.

The source of antioxidants and other bioactive compounds are also microorganisms, including actinomycetes, bacteria [78], cyanobacteria, fungi, and lichens [79]. Compared to plants, these organisms can grow very quickly under strictly controlled conditions, which makes them a favorable source of natural bioactive molecules for industrial food, pharmaceuticals, nutraceuticals, and agricultural applications.

Antioxidants can also be delivered to the body in the form of dietary supplements. The synthetic forms of antioxidants are bioequivalent to their natural forms, e.g., biovitamin C vs. chemically synthesized L-ascorbic acid, or synthetic and natural R, R, R-α-tocopherol. Antioxidants are also used as additives to prevent the oxidation of unstable ingredients in the food, cosmetic, and pharmaceutical industries. This mainly concerns synthetic antioxidants with a phenolic structure, such as butylated hydroanisole (BHA), butylated hydrotoluene (BHT), and tert-butylated hydroquinone (TBHQ), which are added to foodstuffs to prevent lipid rancidity [80].

Antioxidants differ in their ability to scavenge free radicals. It has been shown that antioxidant activity can be significantly correlated with the number of active groups such as OH or NH_2_ and the position of these functional groups in the order ortho > para > meta, from the highest to the lowest active [81]. It should be remembered that antioxidants can act through various mechanisms, not only scavenging radicals, but also sequestering transition metal ions, decomposing hydrogen peroxide or hydroperoxides, quenching active pro-oxidants, and enhancing endogenous antioxidant defense but also by repairing the resulting cellular damage. Therefore, antioxidants are sometimes classified as primary or chain-breaking antioxidants and as secondary or preventive antioxidants [82]. Primary antioxidants actively inhibit oxidation reactions by scavenging ROS/RNS, while secondary antioxidants act indirectly through chelation of transition metal (iron) ions [83,84] and other specific actions such as anti-inflammatory, induction of protective factors, inhibition of NADPH oxidase (nicotinamide adenine dinucleotide phosphate oxidase), inhibition of xanthine oxidase, and regulation of redox-sensitive signal transduction pathways, including transcription factors and inhibition of poly (ADP-ribose) −1 (PARP-1) polymerase [81,85,86]. Another indirect way of antioxidant activity is the activation of transcription factors, including Nrf2, which in turn leads to the activation of endogenous antioxidant enzymes [87].

Currently, the role of exogenous antioxidants in preventing or delaying oxidative damage is becoming more and more controversial. The initial enthusiasm for their positive health effects was mainly based on in vitro experiments. In the initial studies, the in vivo bioavailability of the antioxidants, which is generally quite low, was neglected. In this context, the activity of scavenging free radicals by antioxidant metabolites seems to be more reliable [50,88]. The high in vitro chemical reactivity of the antioxidant is therefore not evidence of its effectiveness in vivo. Moreover, as shown by individual studies [89,90], supplementation with antioxidants may be ineffective and even very dangerous. An example may be the disappointing research on the effectiveness of vitamin E in the risk of cardiovascular disease or hemorrhagic stroke [91,92,93,94,95]. Reports that the use of antioxidants not only prevent cancer but may also provoke it are also alarming [96]. As it turns out, it is especially dangerous to supplement with antioxidants in doses exceeding the daily intake. For example, supplementation with β-carotene over ten times the daily intake increased the incidence of lung cancer in smoking men by 18% [97]. Vitamin C supplementation is particularly controversial. Linus Pauling recommended health-promoting use of a high daily dose of 1000 mg [98]. Unfortunately, it turned out that even at low concentrations of ascorbic acid, a pro-oxidative effect can occur in the presence of transition metals, e.g., iron. An example of this effect is the effect of ascorbic acid on iron-induced lipid peroxidation [99].

In the review by Hrelia and Angeloni [100], recent reports on new mechanisms of action of natural antioxidants are collected. Their study highlights the fact that natural antioxidants are heavily metabolized in vivo, a result of which is that their redox potential drops significantly at the physiological level.

The authors observed a growing interest in the scientific community in the interactions of natural antioxidants with proteins that are involved in intracellular signaling cascades and modulation of the gut microflora.

Currently, in research on natural antioxidants, research issues can be distinguished regarding (i) combination therapies using the synergistic effect of natural antioxidants, (ii) anti-aging effects of fermented preparations, (iii) enzyme research, (iv) genetic research, (v) studies on the effect of antioxidants on the intestinal microflora, and (vi) the effect of antioxidants on hormonal activity.

## 3. Antioxidant Capacity/Activity Measurements

Determination of antioxidant status attracts growing attention for clinical purposes [48,101]. However, the determination of antioxidative potential, in this case, is difficult to establish due to the complex mechanisms of action for the individual anti-oxidants. Some of them act by scavenging free radicals, some by preventing the formation of ROS or inducing the signaling pathways or by repairing the oxidative damage. Cellular protection is ensured mainly by enzymes (glutathione peroxidase, SOD, catalase), whereas the non-enzymatic antioxidants act in the plasma. Additionally, the status of redox homeostasis differs significantly between the individuals; therefore, the reference values have not been established so far [102,103]. Presently, there is also no direct method dedicated to accurate measurement of oxidative stress in vivo conditions. Therefore, oxidative stress is measured by the use of multiple in vitro assays [102], which can identify free radicals directly like electron paramagnetic resonance (EPR) or electron spin resonance (ESR) spectroscopy, fluorescent probes, or indirect methods enabled to identify the stable products which are created as a consequence of the free radical attack, like chromatography, colorimetry, and immune, or enzymatic tests [104].

There is also some misunderstanding regarding specific terms that are used to describe antioxidants measurement assays. Bunaciu et al., in a critical review [105], pointed out that the terms “antioxidant activity” and “antioxidant capacity” need some more clarification because they are often used interchangeably despite having different meanings. It should be emphasized that the term “antioxidant activity” refers to kinetic-based assays measuring the rate constant of a reaction between reactants or scavenging percentages per unit time. Thus, the term is characteristic of a specific antioxidant and oxidant, expressed as reaction rates value. In turn, the antioxidant capacity can be defined as the efficiency of antioxidants to inhibit the oxidative degradation of the various bio-compounds. The measurements are based on the reaction between studied antioxidants and free radicals (reactive species inactivation, quenching, or scavenging) or on the reaction of the sample with transition metals. Antioxidant capacity expresses the amount (in moles) of a given free radical that is scavenged by a sample.

In the case of a heterogeneous mixture, the antioxidant capacity of each individual component is not possible to measure as all antioxidants react simultaneously to produce the total scavenging ability of the sample. In the case of the complex samples, the most reasonable way of their antioxidant capacity is using a variety of methods that can address the different mechanisms of action of individual components [106,107]. The collaborative effect of all sample components (i.e., synergistic or antagonistic effects) is responsible for “total antioxidant capacity” (TAC) measured.

Antioxidants’ capacity can be estimated by considering the final effects of their presence, by the use of in vitro tests, or directly by more complex methods utilizing exogenic probes to detect oxidation. With such a variety of mechanisms involved in the action of antioxidants, determining the level of total antioxidant capacity (TAC) is one of the major challenges in antioxidant testing. Thus far, no universal method has been developed that would gain general and univocal acceptance. Therefore, when choosing a specific method, one should be aware of what kind of an antioxidant function is being measured [46,108].

The measured activity of primary antioxidants reflects their ability to scavenge ROS/RNS throughout hydrogen atom (H^•^) or electron (e^−^) transfer or both species simultaneously (i.e., proton-coupled electron transfer). Secondary antioxidants, which are known as preventive ones, are evaluated by the chelating ability of selected transition metal ions e.g., Fe(II) or Cu(I). Preventive antioxidants act by inhibiting Fenton reactions as a source of hydroxyl radicals or a Lewis acid-base neutralization (metal ion—antioxidant). In turn, endogenous antioxidative enzymes, being “first-line defense antioxidants” such as SOD, CAT, and GPx, which are able to scavenge superoxide anion radicals and hydrogen peroxides, require enzymatic methods for evaluation of the antioxidants activity [108].

**Figure 2:**
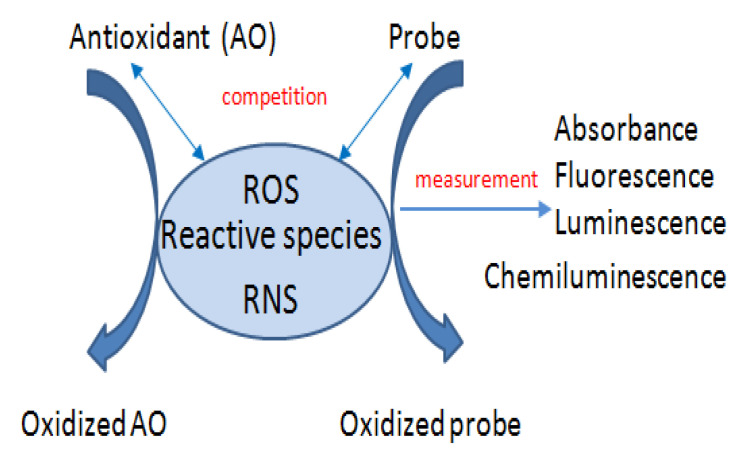
Schematic illustration of competitive antioxidant (AO) assay.

Nonenzymatic primary antioxidant assays can be non-competitive or competitive [109]. Competitive assays such as TRAP, ORAC, TOSC, crocin bleaching, peroxyl radical trapping antioxidant parameter, act due to the competition between a fluorogenic or chromogenic probe and antioxidants for the reactive species (ROS/RNS). In the presence of antioxidants, the probe undergoes weaker oxidation, which is reflected in the changes of its measurable properties (absorbance, fluorescence, luminescence) [110] (Figure 2).

**Figure 3:**
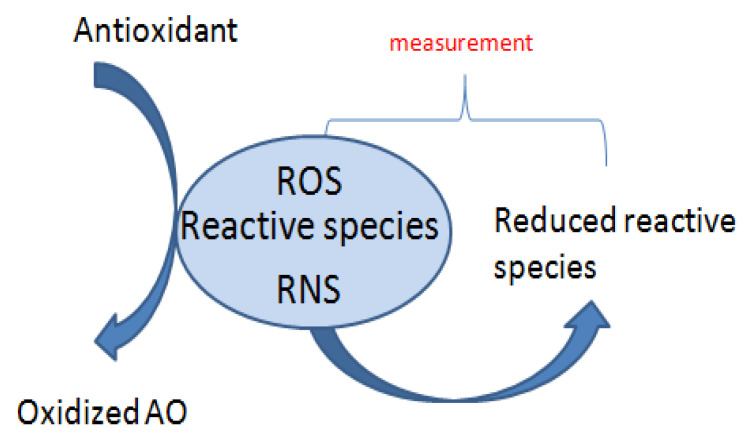
Schematic representation of non-competitive antioxidant (AO) assay.

The non-competitive (Figure 3) ones based on Folin−Ciocalteu reaction, ABTS/TEAC, CUPRAC, FRAP, DPPH, ABTS differ in the lack of the presence of any competing target molecule. TAC measurements are considered to be noncompetitive if they rely on electron transfer (ET) mechanism, whereas competitive measurements are usually based on a hydrogen atom transfer (HAT) [46].

In certain circumstances, ET/HAT mechanisms may not be easily identified like for 2,2′-azinobis-(3-ethylbenzothiazoline-6-sulfonic acid (ABTS), and 2,2-diphenyl-1-picrylhydrazyl (DPPH) assays, which are sometimes classified as mixed-mode assays (ET/HAT). Both free radicals react according to two mechanisms: HAT (1) and SET (single electron transfer) (2):

HAT: DPPH^●^ + A → DPPH-H + A^●^, (1)

SET: DPPH^●^ + AH → DPPH^−^ + AH^+●^; AH^+●^ → AH^●^ +H^+^; DPPH^−^ + H^+^ → DPPH-H, (2)

Experimental investigations [111,112] confirm that HAT and SET transformations may occur at the same time as a sequential proton-loss electron transfer (SPLET), which is also named as a proton-coupled electron transfer (PCET) [106]:

SPLET: AH → AH^−^ + H^+^; AH^−^ + DPPH^●^ → AH^●^ + DPPH^−^; DPPH^−^ + H^+^ → DPPH-H (3)

It has been proven that the HAT mechanism dominates in aqueous solutions. In turn, the SET and SPLET may dominate in non-aqueous solutions due to the possibility of organic solvents forming hydrogen bonds with molecules of antioxidants [113,114,115,116]. Among the SET methods, the most used are DPPH radical scavenging capacity assay, Trolox equivalent antioxidant capacity (TEAC or ABTS) assay, ferric reducing (FRAP) assay, reducing power assay (RP), and copper reduction (CUPRAC) assay. HAT assays include the total per-oxyl radical-trapping antioxidant parameter (TRAP) assay, the crocin bleaching assay, oxygen radical absorbance capacity (ORAC) assay, and total oxyradical scavenging capacity (TOSC) assay.

Antioxidant activity can also be estimated using nanoparticle-based assays utilizing nanoparticles probes exhibiting localized surface Plasmon resonance (LSPR) absorption [117,118]. It has been established that the LSPR absorption connected with the nanoparticles grove rises linearly depending on antioxidant concentration. Scampicchio et al. described such correlation for gold nanoparticles (AuNPs) generated under the influence of phenolic acid antioxidants being able to donate electrons. Özyürek et al. proved the same for silver nanoparticles (Ag-NPs), which were formed as a product of AgNO_3_ reduction with polyphenolic antioxidants.

Many studies are dedicated to the estimation of the antioxidant power of various individual chemicals, as well as food samples and natural extracts [119]. For this purpose, various tests were applied, including, among others, the oxygen radical absorbance capacity test, the Trolox equivalent antioxidant capacity, and the ability to reduce metal ions, such as copper or iron. Several reviews have been published that highlight the advantages and disadvantages of the available tests [120,121,122,123,124]. However, there is still no standard quantitative method for measuring antioxidant activity. Therefore, it is extremely difficult to compare the results obtained from different studies. The complexity and variety of research systems make it impossible to repeat and confirm experiments by independent laboratories. The most common methods related to the antioxidant assessment are summarized in Table 2.

### 3.1. Techniques Used to Assess Antioxidant Capacity

A number of techniques are used to assess antioxidant capacities, such as UV-Vis spectroscopy, fluorescence spectroscopy, chemiluminescence, electron paramagnetic resonance (EPR), enzyme-catalyzed assays [164,165,166,167,168], and cell culture assays. Moreover, there are some electrochemical techniques, including controlled potential techniques, electrochemical sensors, and biosensors, which are commonly applied [169]. However, the most widely used techniques for evaluating the ability of an antioxidant to scavenge e.g., ABTS^●+^, DPPH^●^, O_2_^●−^, H_2_O_2_, a total antioxidant reducing capacity, e.g., TEAC, ORAC, and FRAP belong to spectrometric techniques. These methods have been commonly used to determine the antioxidant capacity of many plant extracts, foods, and dietary supplements [170,171,172,173,174]. These assays despite some drawbacks [129] are easy to use.

#### 3.1.1. DPPH Free Radical Scavenging Assay

**Figure 4:**
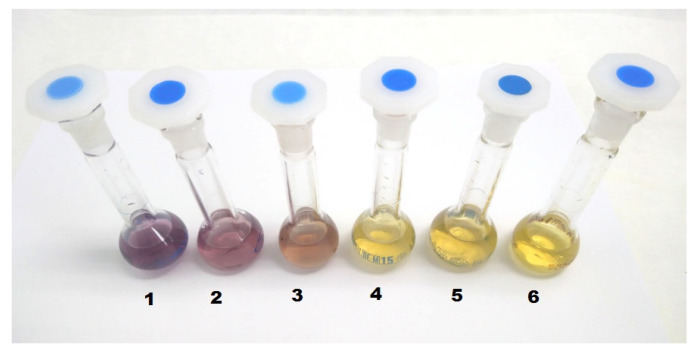
1 mM DPPH solutions containing an increasing amount of Salvia officinalis extract: 1–20 μL, 2–30 μL, 3–50 μL, 4–80 μL, 5–100 μL, 6–120 μL.

To measure antioxidants’ power, their ability to deactivate free radicals was used. One of the most frequently used stable free radicals is DPPH (1,1-diphenyl-2-picrylhydrazyl) discovered by Goldsmith and Renn in 1922 [175]. Due to the relocation of the unpaired electron, DPPH forms a stable radical cation and does not form dimers in alcohol solutions [176,177]. The DPPH solution has a dark purple color with maximum absorbance at wavelength = 517 nm. By reaction with a substance that gives off a hydrogen atom, a reduced form of DPPH 2,2-diphenyl-1-picrylhydrazine is formed, and then the purple color of the solution changes to yellow with a concomitant decrease in absorbance (Figure 4).

The drop in absorbance is proportional to the amount of DPPH oxidized form that remains in solution. The color change from purple to yellow can be monitored spectrophotometrically and utilized for the assessment of the free radical scavenging potential of many antioxidants and natural products. For the first time, the colorimetric method was described by Blois [177] for the evaluation of the antioxidant properties of the thiol-containing amino acid cysteine as the model antioxidant. Since that time, an easy and convenient colorimetric method has been extensively used to evaluate the antioxidant capacity of many products of natural origin [178,179,180,181,182,183]. The reaction of DPPH with antioxidants was adapted for illustration and measuring the kinetics of radical quenching [184,185]. Since the beginning of the 1960s, the method, as well as antioxidant activity calculations, have evolved into numerous modifications [186,187].

##### DPPH Free Radical Scavenging Kinetics

DPPH free radical scavenging has been conducted by using at least two commonly practiced procedures (a) fixed reaction time, when the researcher imposes reaction times of 15, 30, or 60 min, and (b) steady-state saturation one, when the reaction time is related to the reaction kinetics. The reaction of DPPH radicals with antioxidants is a kinetically driven process. It has been proven that the time required to reach saturation state, i.e., the highest decrease in DPPH absorbance depends on concentration and the kind of antioxidant. To check out the kinetic behavior of the disappearance of DPPH radicals with individual antioxidants, kinetic scans should be performed at different concentration levels. Although at higher concentrations, the scavenging capacity is higher, sometimes the reaction cannot be completed quickly because of slow kinetics. For instance, the reaction of DPPH with ascorbic acid is fast and achieves completion within a minute [188], whereas even 3 h is not enough to finish the reaction for curcumin at so small a concentration as from 5 to 15 µM. In turn, the reaction time for BHT was found to be around 6 h. Such antioxidants as lipoic acid, melatonin, and pentoxifylline demonstrate slow reaction with DPPH radical up to 2 mM. Such kinetic measurements have been performed for different chemicals used as reference antioxidants. Considering the time duration of reaction to achieve the steady-state, antioxidants can be divided into categories of fast (<30 min), medium (30 min to 1 h), and slow (>1 h) kinetics. In 2012, Mishra et al. [178] established the nature of individual chemicals such as alpha-tocopherol, ascorbic acid, sesamol, gallic acid, ferulic acid, and BHT-butylated hydroxytoluene, which are commonly used as references in the comparative evaluation of antioxidant properties. Among these reagents, there are examples of fast (ascorbic acid), medium (gallic acid), and slow reaction kinetics, which is observed for BHT. Despite the fact that the time to attain an equilibrium state depends on the nature of antioxidants, researchers have usually chosen a fixed reaction time mode where reaction time is pre-imposed to be 20–30 min instead of the real-time required to attain completion of the redox reaction [176], ignoring their kinetic behavior and the fact that many antioxidants might react with different kinetics or might not react at all. Furthermore, some authors emphasize the reversibility of the free radical reduction by antioxidants, which results in underestimation of the antioxidant capacity of many antioxidants [106,189].

Considering numerous methodologies of DPPH assay described in the literature, involving variation in (i) concentrations of reagents, (ii) sample’ volume, (iii) the kind of reference molecules, (iv) antiradical parameters used, (v) units of applied parameters, and (vi) the kind of sample environment (methanol or semi-aqueous media), the antiradical potential of any sample assessed by DPPH assay, it is very difficult to compare results between laboratories. Mishra et al. [134] collected IC_50_ values of reference standards such as butylated hydroxyl anisole (BHA), ascorbic acid, gallic acid, BHT, and Trolox that determined by different authors. It appeared that the reported IC_50_ value of ascorbic acid was in the range from 11.85 to 629 µM. Unfortunately, such a large variation in IC_50_ values was also observed for remaining antioxidants. Recently, Xie and Schaich [190] have reevaluated the DPPH assay considering the solvent kind and pH values.

##### Parameters Used to Express the Antioxidant Potential

The DPPH free radical scavenging activity is commonly expressed in terms of the percentage of inhibition of the free radical by examined antioxidants. The EC_50_ value relates to the antioxidant concentration required to achieve a 50% decrease in the DPPH absorbance. This parameter is typically employed not only to express the antioxidant capacity but also to compare the activity of different compounds with each other. To find the above parameter, antiradical curves are plotted, representing the relationship between the concentration of antioxidants on the x-axis and relative scavenging capacity (E%) on the y-axis. The radical scavenging capacity can be calculated using the following equation:

(4) $$\mathrm{Percentage}\;\mathrm{effect}\;\left(E\%\right)=\frac{\left({\mathrm{Abs}}_{\mathrm{control}}-{\mathrm{Abs}}_{\mathrm{sample}}\right)}{{\mathrm{Abs}}_{\mathrm{control}}}\times 100\%$$

However, to find the most credible EC_50_ value, an assay should be done using several antioxidant concentrations located near the estimated ED_50_ value. The above graph looks like a typical rectangular hyperbole, but it can be changed into a sigmoidal curve after the logarithmic transformation of the x-axis (log[mol/L]). The EC_50_ value is usually located in a short linear range, and it may be calculated by the use of the right-angled triangle [191,192]. This mathematical method must meet two assumptions: reaching the maximum response and recording at least two points located near the targeted point of the 50% maximal response. The following equation enables EC_50_ value calculation:

(5) $${\mathrm{EC}}_{50}=D-\frac{\left(A-50\%\max.\mathrm{response}\right)\times \left(D-C\right)}{\left(A-B\right)}$$

It should be noted that sigmoid curves based on the Hill equation are easier to interpret [193]. The logarithmic curve does not have to be symmetrical around its midpoint, thanks to the model using the Richards equation which provides a fitting thanks to the introduction of the S parameter, quantifying the asymmetry. Chen et al. [192] conducted a comparative study of several specialized computer programs based on various regression models towards the aim of EC_50_ estimation. The EC_50_ values obtained by the use of the statistical programs were similar to each other; however, GraphPad Prism@ five-parameter analysis showed the smallest variance in relation to the experimental estimated EC_50_. The authors claim that the observed differences in the results between the statistical processing programs GraphPad and SigmaPlot are due to the fact that the first one calculates actual EC_50_ values, while the second gives the inflection point as the EC_50_.

Antiradical power (ARP) is another parameter that can be used to define antioxidant activity. This parameter is defined as a reciprocal of EC_50_, which is why the higher value of EC_50_ is related to smaller antiradical power:

(6) $$\mathrm{ARP}=\frac{1}{{\mathrm{EC}}_{50}}$$

The antioxidant capacity can be expressed as reference chemical equivalent such as Trolox (µmol TE/g), ascorbic acid, gallic acid (GAE/g), etc. Unfortunately, comparison of results presented by different studies is difficult because of the variety of units used for the above recalculations. We can find mass/mass units such as milligrams per gram of dry material, µmol/g, or mass/volume ones.

##### DPPH Assay Approaches

In the original DPPH assay, provided by batch experiments, several automation approaches based on flow injection analysis (FIA) [194,195] and sequential injection analysis (SIA) [196] have been proposed in recent decades. An interesting approach inspired by HPLC-FIA [197] has been elaborated on by Koleva [198]. In this method, the HPLC-separated analytes react postcolumn with the DPPH solution, and the induced bleaching is detected as a negative peak by the second detector at 517 nm. Cerda et al. [199] described multi-syringe flow injection analysis (MSFIA) for determining the total antioxidant capacity of several food products. Flow injection analysis (FIA), similarly to sequential injection analysis (SIA), is beneficial for rapid testing of antioxidation/radical scavenging activity of large series of multicomponent samples [177]. Another advantage of automatic approaches in comparison to the standard spectrophotometric batch experiments lies in the visible improvement of measurement reproducibility. Another assay suitable for screening of either hydrophilic or lipophilic antioxidants is a high-throughput relative DPPH radical scavenging capacity (RDSC) assay elaborated by Cheng et al. [189]. The assay, which can be performed in aqueous and organic environments, utilizes a 96-well microplate reader with the spectrophotometric detector, ensuring acceptable accuracy, precision, and reproducibility.

The sophisticated instruments are required not only for the rapid determination of the antioxidant activity of complex mixtures but also for providing separation and identification of the selected antioxidant compounds. The HPLC method appears to be the method of choice in this case. For this purpose, HPLC should be used in combination with an appropriate detector, which is usually connected online to chromatographic apparatus. However, simultaneous determining of antioxidant capacity requires additional coupling with another radical scavenging detection mode. Such systems have been described in the literature; unfortunately, they are not adopted commonly due to their complexity and the lack of commercial availability. As an example, in 2007, Wu et al. [200] developed HPLC-ESI-MS and NMR for estimation of antioxidant capacity of polyphenolic acids in the plant extract. In turn, Nuengchamnong et al. [201] proposed RP-HPLC coupled with an electrospray ionization MS/MS system for the identification of antioxidant compounds in an extract of a Thai medicinal plant. An interesting HPLC approach, suitable for searching natural antioxidants in plant extract of Flos Lonicerae Japonicae, was developed by Tang et al. in 2008 [202]. The method’s idea assumes that the peak areas of compounds with antioxidant activity undergo reduction after reaction with DPPH. The authors performed additional identification of antioxidants by the HPLC-DAD-TOF/MS hyphenated technique.

Traditional thin-layer chromatography with post chromatographic derivatization using DPPH solution for free radical scavenging activity evaluation, discovered by Glavind and Holmer in 1967 [203], exists nowadays in the modern version owing to video scanning technology [204].

#### 3.1.2. Electrochemical Methods

Electrochemical measurements possess some major advantages in comparison to spectrophotometric methods mainly due to the fact that they are fast, less tedious, cheaper, and safer for the environment. They include electrochemical techniques of antioxidant characterization as potentiometry, amperometry, biamperometry, cyclic voltammetry (CV), square-wave voltammetry (SWV), and differential pulse (DPV). These methods utilize the fact that antioxidants are involved in redox reactions acting as reducing agents. The electrochemical techniques are able to measure their redox potentials.

##### The Cyclic Voltammetry Method

The cyclic voltammetry method is applied to screen the reducing capacity of the samples. Cyclic voltammetry (CV) operates due to the combination of three electrodes, namely working electrode, reference, and auxiliary electrode. A polarogram representing the relationship between current intensity and an increasing potential applied to the working electrode is recorded. The obtained voltammograms show well-defined voltammetric peaks corresponding to the oxidation and reduction processes. Lower Epa values are associated with the higher reducing activity of the tested sample. Therefore, considering the first oxidation potential, the following classes of chemical compounds can be distinguished: if Ep is lower than 0.8 V, antioxidant power is high, and if Ep is between 0.8 and 1.3 V, antioxidant power is low [205]. The area under the curve of the voltammetric peak (AUC) corresponds to the concentration of antioxidants. Broad anodic peaks are usually observed due to the response of multiple reducing agents with different oxidation potentials present in the respective extracts. In such cases, Chevion et al. [206]. Martinez et al. [207], and Zielińska and Zieliński [208] suggested that the area under the anodic current wave should be used for the evaluation of reducing the power of the samples. Lower AUC indicates a lower reducing capacity of the investigated extract. Usually, the reducing capacity is statistically significantly correlated with the active components of the extracts. Zielińska et al. [209] found the existence of a significant positive correlation between the total phenolic content (r = 0.867; *p* < 0.01) and total flavonoid content (r = 0.752, *p* < 0.01) with the reducing capacity of peels of the investigated apple cultivars.

##### Biamperometry

Determination of the antioxidant activity by biamperometric measurements is based on a high degree of reversibility redox couple potential, including Fe^3+^/Fe^2+^, I_2_/I^−^, Br_2_/Br^−^, VO_3−_/VO_2_^−^ Fe(CN)_6_^3−^/Fe(CN)_6_^4−^, and Ce(IV)/Ce(III). The DPPH^•^/DPPH couple is also suitable for this purpose. The current intensity is proportional to the decreasing concentration of free radicals after reaction with the antioxidants. The obtained results of antioxidant activity are usually in very good agreement with those determined by the use of other conventional methods such as spectroscopic measurements. The biamperometric technique was applied by Milardovic et al. [210] for evaluation of the selected standard antioxidants (ascorbic acid, uric acid, gallic acid, N-acetyl-l-cysteine, glutathione, caffeic acid, ferulic acid, sinapic acid, catechin hydrate, quercetin) and food samples such as coffee, tea, wine, and juices.

#### 3.1.3. Nanoparticle-Based Approach for the Antioxidant Activity Measurement

More recently, the new nanoparticle-based approach for evaluation of antioxidant activity has been reported. This approach utilizes the unique optical, electronic, and catalytic properties of metallic nanoparticles (1–100 nm) [211,212,213,214].

For the first time, Scampicchio et al. [117] described a nanoparticle-based method for measuring antioxidant activity. The idea of the method was based on the catalytic growth of gold (Au) NPs mediated by phenolic acids as active reducing agents (vanillic acid, propyl gallate, protocatechuic acid, caffeic acid, ferulic acid). It appeared that the antioxidant (reducing) power of the phenolic acids was correlated with the optical properties of generated nanoparticles. The absorbance characteristic of the plasmon of the Au NPs (555 nm) was linearly dependent upon the concentration of the investigated phenolic acids. The authors confirmed the good agreement between the total phenolic content estimated by the Folin-Cicolteau spectrophotometric determination and the results of the Au NPs protocol.

**Figure 5:**
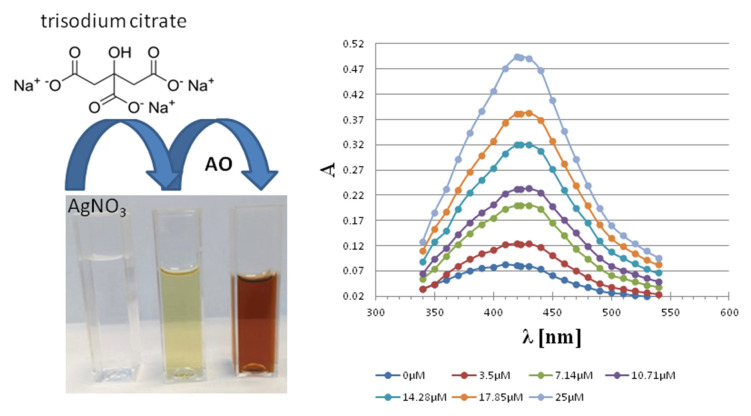
Scheme illustrating the idea of Silver NanoParticle Antioxidant Capacity (SNAPC) Assay. On the left side, sample preparation steps; on the right side, surface plasmon resonance absorption of citrate-stabilized AgNPs. Absorption is intensified by the addition of increasing ascorbic acid (AO) concentration, which corresponds to NPs growth.

A few years later, Özyürek et al. [118] elaborated on a sensitive colorimetric method based on the reduction of Ag+ ions to silver nanoparticles (AgNPs) for the detection of polyphenols. The AgNPs revealed the absorption band at 423 nm, allowing the quantification of the polyphenols. The initial seeds were formed by the reduction of silver ions with trisodium citrate. The addition of antioxidants as secondary reductants caused the reduction of Ag+ ions on silver seeds and the deposition of more Ag atoms on the seeds, resulting in the final core−shell AgNP structures. The growth of AgNPs on monodisperse seed particles caused a linear, concentration-dependent absorbance increase. The method was named by the research group “Silver NanoParticle Antioxidant Capacity”, abbreviated as the SNPAC method, which is recommended for measuring the total antioxidant capacity (TAC) of a wide range of plant samples (Figure 5).

**Figure 6:**
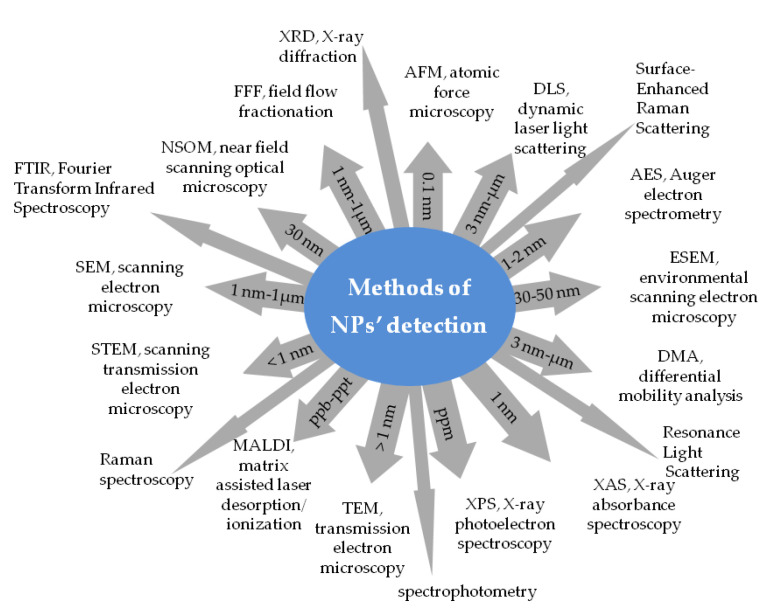
Selected methods applied to detection of nanoparticles together with detection limits [217].

Until now, most assays applied for antioxidant capacity determination have involved the use of NPS of gold, silver, Fe_3_O_4_, quantum dots, and titania nanoparticles. The estimation of antioxidant activity relies on the antioxidant-mediated growth of NPs, monitoring changes in NPs size, changes in surface oxidation states, the degree of agglomeration of nanostructures, and optical monitoring of the plasmon absorption bands. AuNPs are still the most commonly used for that purpose. AuNPs have a very characteristic absorbance peak at 517 nm. AuNPs are soluble and stable in different solvents such as water, dichloromethane, or methanol. NPs formation can be monitored visually owing to AuNPs’ color, which depends on their shape and size, but also surface-adsorbed species, the refractive index of the dispersion medium, and interparticle interactions [215]. Different techniques have been engaged for detection and characterization of NPs such as the localized surface plasmon resonance (SPR), Surface-Enhanced Raman Scattering, spectrophotometry, Fourier Transform Infrared Spectroscopy (FTIR), Resonance Light Scattering, Raman spectroscopy, X-ray diffraction (XRD), and transmission electron microscopy (TEM) [216]. Selected methods suitable for measuring size, electric and mechanical properties, size distribution, hydrodynamic radius, elemental composition, and quantitative analysis of nanoparticles together with the methods’ detection limits are illustrated in Figure 6.

Since nanoparticles-based assay is a new analytical tool, calibration is usually performed using control antioxidants [218,219,220,221], and additionally, the assay is compared with reference methods, e.g., ORAC and TEAC. Many authors achieved very good agreement between the TAC values obtained by the nanoparticle-based approach and the Trolox Equivalent Antioxidant Capacity (TEAC), CUPRAC [222], Folin-Ciocalteu, FRAP, and DPPH [218] as reference tests.

Antioxidant capacity determination by nanoparticles-based method also involves other metallic or metal oxide NPs. Gatselou et al., in 2016 [223], reported that phenolic compounds (i.e., gallates, catechins, dihydroxybenzoic acids, and cinnamates) generate changes in the localized surface plasmon resonance of rhodium NPs, causing characteristic spectral and color transitions in their suspensions. Under the influence of the reaction between phenolic compounds and rhodium, absorbance at 450 nm and 580 nm increased linearly together with increasing concentration of antioxidants in the range of 0–500 μM.

Recently, antioxidant activity (AOA) assays using cerium oxide nanoparticles (CeO-NPs) as a novel colorimetric sensor were developed. Cerium oxide nanoparticles (CeO-NPs) may act as both an oxidant and an anti-oxidant, switching between trivalent and tetravalent oxidation states [224]. In 2018, Ozdemir Olgun [225] elaborated on a novel colorimetric sensor consisting of the poly(acrylic acid) sodium salt (PAANa)-coated CeO-NPs which oxidized a peroxidase substrate, namely tetramethyl benzidine (TMB) in acidic conditions to charge-transfer complex of a blue color. The analytical wavelength of the colored product was estimated at 651 nm. The antioxidant activity evaluation was based on the measurement of decreasing intensity of the nanoceria suspension absorbance caused by antioxidants. The authors demonstrated that the antioxidant capacities of hydrophilic and lipophilic antioxidants such as rutin, tetramethyl benzidine, quercetin, ascorbic acid, Trolox (6-hydroxy-2,5,7,8-tetramethylchroman-2-carboxylic acid), ferulic acid, BHT, caffeic acid, and catechin estimated by the above procedure were compatible with those of reference assays ABTS, CUPRAC, and CERAC [225]. Currently, portable nanoparticle-based tests for rapid detection of food antioxidants (NanoCerac) are being developed, e.g., for nanoparticles of immobilized cerium oxide [226], or nanoparticles of metal oxides TiO_2_, Fe_2_O_3_, ZrO_2_, ZnO, and SiO_2_, which are immobilized on cellulose [227]. Several reviews regarding TAC determination by using NPs can be found in the literature [228,229,230,231,232].

## 4. Antioxidant Capacity of Extracts from Natural Sources

Epidemiological research confirms that the conditions related to oxidative stress can be improved by the consumption of food products rich in numerous compounds with high antioxidant activity [233,234]. Natural products containing at least 0.1% of antioxidants can be accepted as dietary supplements with antioxidant properties.

As the total antioxidant capacity (TAC) covers the additive (synergistic/antagonistic) action of different antioxidants of complex samples, most researchers use this parameter to assess plant-based extracts rather than the separate determination of the concentrations of the individual constituents. It should be emphasized that antioxidant capacity reflects the thermodynamic conversion efficiency of reactive species by antioxidants in contrast to the antioxidant activity, which is related to the kinetics of this reaction, usually expressed scavenging percentages per unit time. Unfortunately, many phytochemical studies have reported conflicting results, which is why TAC assays still require consideration and standardization in the following issues: (i) procedures of sample preparation, (ii) expressing results, (iii) statistical validation (e.g., using certified reference compounds that take into account the different reaction kinetics), and (iv) establishing effects of solvent, concentration, pH, etc.

The choice of extraction techniques has the greatest impact on the composition and concentration of the bio-composition of both active compounds and matrix components obtained from a wide range of plant materials (herbs, vegetables, berries, and fruits) [235]. It has been shown that different extraction methods lead to different extraction yields on the same plant material [236]. For example, Lisitsyn et al. [237] studied the plant extracts (rosemary, black pepper, thyme, and sage) obtained by the use of supercritical CO_2_ extraction. Owing to this extraction method, they produced extracts with a significantly different composition in comparison to those obtained in traditional ways. It appeared that supercritical extracts were rich in a variety of substances with high antioxidant, and antimicrobial activities such as alkaloids, terpene, phytosterols, waxes, pigments, high molecular weight unsaturated and saturated fatty acids, and vitamins.

Currently, classic extraction techniques, i.e., Soxhlet extraction, maceration, percolation, and distillation, which use large amounts of volatile organic solvents, or elevated temperature, are less frequently used due to the requirements of the so-called “green chemistry”, poor efficiency, and possible thermolability of extracted analytes [238]. High extraction efficiency and effectiveness are possible thanks to the use of unconventional techniques, i.e., Solid Phase Microextraction (SPME), Supercritical Fluid Extraction (SFE), Microwave-Assisted Extraction (MAE), Pulsed-Electric Field (PEF) Extraction, Ultrasound-Assisted Extraction (UAE), and Enzymatic Treatment or Pressurized Liquid Extraction (PLE). It should be remembered that antioxidant capacity changes not only in relation to the extraction techniques but varies with the growth period and drying methods and between plant parts. These factors’ influence has been confirmed by a multivariate analysis performed by Buitrago et al. [239] using *Chenopodium quinoa* Willd.

Plant-derived compounds possess well-known and established antioxidant activity. However, the microbes are also efficient producers of primary and secondary metabolites with specific antioxidant potential [240]. Thus far, microbial metabolites have been recognized as efficient remedies against fungal and bacterial infections (tetracyclines, amphotericin, penicillins, erythromycins, streptomycin, and vancomycin), cancer (daunorubicin, bleomycin, mitomycin, doxorubicin,), transplant rejection (rapamycin, cyclosporine), or high cholesterol (mevastatin, lovastatin) [241].

Almost all eubacteria possess the ability to produce a variety of extracellular metabolites with significant antioxidant activity, such as thiazostatins A, phenazoviridin, (Z)-1-((1-hydroxypenta-2,4-dien-1-Yl)oxy)anthracene-9,10-dione, 5-(2,4-dimethyl benzyl) pyrrolidin-2-one, benthophoenin, benzastatins C, benthocyanins A, B, C, exopolysaccharides (EPS), and benzastatins A [78]. Exopolysaccharides (EPS) are produced by numerous strains of microorganisms belonging to the genera *Lactobacillus*, *Leuconostoc*, *Lactococcus*, and *Streptococcus*, which are abbreviated LAB (Lactic Acid Bacteria) [54]. EPS are characterized by the presence of reactive functional groups including aldehyde, hydroxyl, and ketone groups. They can efficiently react with free radicals. These compounds have a polymeric structure. They are made up of repeating subunits of connected carbohydrates α- and β-glycosidic bonds. Homopolysaccharides are composed of one type of simple sugar, i.e., glucose or fructose, while heteropolysaccharides are more complex. The structure of individual heteropolysaccharides produced by particular species and strains of these bacteria can significantly vary. A common feature of most of them is occurrence in the composition of sugars e.g., rhamnose, arabinose, mannose, xylose, fructose, glucose, and galactose, in various ratios [242]. The EPS may exist in two forms: a cell-bound exopolysaccharide (c-EPS) that strongly binds to the bacterial surface and a released exopolysaccharide (r-EPS) that can be released into the medium [243].

Cyanobacteria and blue-green algae are sources of a significant amount of free radical scavengers such as carotenoids, phycocynanin which are water-soluble pigments possessing N-H reactive groups. Astaxanthin produced by microalga Haematococcus pluvialis possessing several times higher antioxidant activity in comparison to vitamin E [244,245]. Algae are able to produce also other phenolic compounds with reactive OH moieties responsible for an antioxidant activity like carrageenan, bromophenol, fucophlorethols, galactan sulphate, phlorotannins, fucoxanthin, phycoerythrin, shinorine, catechin, por-phyran, epicatechin, gallate, laminaran, vitamin A, alginic acid, phloroglucinol, eckol, fucodiphlorethol G, 7-phloroeckol, dieckol, phlorofucofuroeckol A, 6,60-bieckol, 2,70-phloroglucinol-6,60-bieckol, and triphlorethol-A, [246,247,248]. As a potential antioxidant, phycobilins rich with groups i.e., N–H, COOH, C–O, and O–H produced by cyanobacteria have been described. However, efficient extraction and purification are required for the recovery of phycobiliproteins on an industrial scale [249]. In 2019, the Special Issue of *Antioxidants* focused on recent investigations concerning marine algal antioxidants and specific antioxidant networks functioning in algae [248,249,250,251,252,253,254,255,256,257,258,259].

Lichens produce various extracellular, secondary metabolites that can be used as potential sources of natural antioxidants [260]. At the beginning of the 21st century, the significant free radical scavenging activity of *Cetraria islandica* aqueous extracts [261] and *Usnea ghattensis* [262] methanolic extracts of *Platismatia glauca*, *Parmelia saxatilis*, *Ramalina pollinaria*, *Umbilicaria nylanderiana*, and *Ramalina polymorpha* [263] were described. Fernández-Morianoet et al. [264] prepared a systematic review concerning the key antioxidant compounds in lichens extracts. It appeared that flavonoids and phenols are mainly responsible for the antioxidant activity of the examined extracts [265,266,267,268]. Some of them also exhibited beneficial antimicrobial and anticancer activities [269,270].

Actinomycetes also produce chemically diverse and pharmaceutically useful compounds with antifungal, antibacterial, diabetogenic, antiviral, immunosuppressive, antiparasitic, antitumor, insecticidal, antioxidant, anti-inflammatory, enzyme inhibitory, and others [271]. Actinomycetes originating from different habitats usually manifest very different antioxidant activity [272,273,274,275,276,277]. Published studies show that nitrogen-containing metabolites, such as the carbazole and phenazinylhetero cycles, constitute the main group of antioxidant compounds produced by *Streptomyces spp*. Stealthins contain OH, NH, and CO groups isolated from *S. Aeriouvifer*, *S. Violaceus*, and *S. viridochromogenes* showed even several dozen times stronger activity than vitamin E [278].

Other examples of antioxidant capacity assessment of the different extracts from plants, lichens, fungi, algae, and actinomycetes are collected in Table 3.

## 5. Synthesis of Nanoparticles (NPs) by Natural Extracts

The various types of metallic/metal oxides nanoparticles composed of silver, gold, platinum, palladium, cerium, copper, nickel, selenium, or iron have been described in the literature. Their unique physicochemical properties make them advanced materials for industry and biomedical applications [321]. There is also evidence that, besides natural extracts, some nanoparticles such as carbon nanotubes, metal, and metal oxides, and various types of polymer-loaded nanoparticles also possess antioxidant activity and can scavenge the reactive nitrogen and reactive oxygen species (RNS/ROS) [322,323]. The iron nanoparticles (INPs), due to high catalytic activity, low toxicity, high magnetism, and microwave absorption ability [324,325,326], have already found varied applications in pharmacy (drug delivery), clinical diagnostic (magnetic targeting, negative MRI contrast enhancement, pigments, stem cell sorting), therapy (gene therapy), and analytical chemistry (bio-separation), bioprocesses (environmental remediation, food preservation), industry (lithium-ion batteries) [247,327].

**Figure 7:**
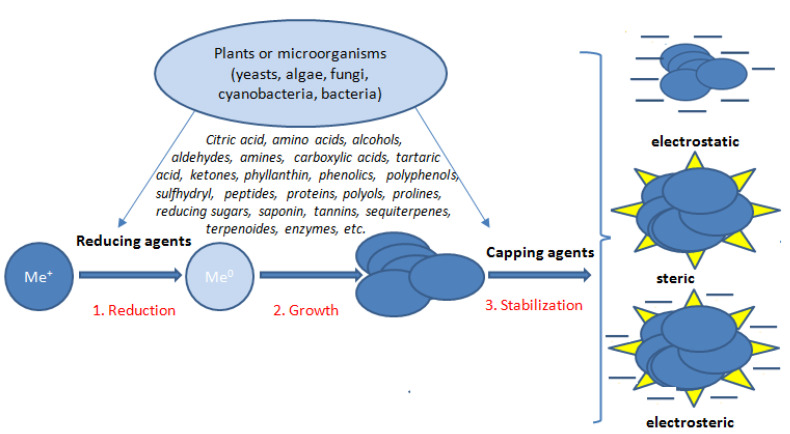
Schematic diagram illustrating the mechanisms of the biogenic synthesis of metallic nanoparticles.

Unfortunately, nanoparticles synthesized by chemical methods often require toxic reducing and stabilizing agents. These toxic substances adsorbed on the surfaces of the nanoparticles limit their applications in biomedical fields [143]. Thus to obtain nanomaterials, the natural synthesis methods involving the reduction of metallic cations by plant extracts, yeasts, fungus, and bacteria are used more and more often. The formation of NPs is achieved via two steps: in the first one, metal ions are reduced, and in the second one the agglomeration of colloidal suspension causing the formation of the oligomeric clusters [328]. So-called “green synthesis” or “biogenic synthesis” has gained more and more attention as an eco-friendly approach useful for synthesis of not only metal/metal oxide nanoparticles but also the production of other nanomaterials, such as hybrid materials, or a variety of bioinspired materials. Nanoparticles produced by green synthesis methods may be less stable compared to nanoparticles obtained as a product of chemical synthesis [329,330,331]. The stabilization of nanoparticles is mainly achieved by electrostatic repulsion. Unfortunately, this type of stabilization is only effective with low ionic strength extracts where the repulsion is facilitated by the highly dispersed double layer. In the case of high ionic strength, aggregation occurs under the influence of strong van der Waals interactions [332]. Another type of stabilization is the creation of an additional barrier on the surface of the NPs. Steric stabilization is provided by proteins, if they are components of the extracts, or by coating the surface with polymers such as PEG or PVP (polyethylene glycol, polyvinylpyrrolidone). Steric stoppers, thanks to their hydrophilic properties, provide an additional stabilizing element in the form of short-distance repulsive forces. The stages of NPs formation and stabilization are schematically illustrated in Figure 7.

Presently, one can observe an increasing interest in studies on the reactivity of nanoparticles compared to macroscopic objects and their cytotoxicity [333,334,335,336,337] accumulation in the body, which can generate reactive oxygen species (ROS) [338,339,340,341]. A relatively new area of research is the use of nanoparticles with redox-active potential as radical scavengers. For example, cerium and yttrium oxides either act as antioxidants [342] or can prevent the increase of ROS [343,344] by mimicking the activity of the oxidative enzymes, catalase, or superoxide dismutase [345]. It has been proven that silver nanoparticles (AgNPs) inhibit cell proliferation and modulate the activity of antioxidant enzymes [340,346,347]. Hirst et al. confirmed by an in vivo test on mice the effectiveness of cerium oxide nanoparticles (CONPs) in treating oxidative stress [348]. A comparative study conducted by Caputo et al. (2015) revealed that the antioxidant potential of N-acetyl-cysteine and Trolox (soluble analogues of vitamin E) was significantly lower in comparison to CONPs [349]. The authors highlighted the CONPs regenerative redox cycle influencing the stability of the antioxidants molecules.

### 5.1. Microbial Synthesis of NPs

The first experiments on AgNPs biosynthesis using bacteria were carried out in the culture of Pseudomonas stutzeri AG 259, Morganella sp. Bacillus subtilis [350]. Using microscopic and spectral techniques SEM, TEM, EDX, and EDS, it was possible to identify various shapes of nanoparticles, i.e., triangular, hexagonal, and spherical with sizes ranging from a few to several hundred nm. The synthesis process was initiated within the first hour of cultivation. The obtained NPs were coated with protein, which allowed them to maintain stability and avoid their aggregation. It has been shown that the enzyme nitrate reductase is responsible for the reduction of silver ions. Thus far, the participation of other groups of enzymes, whose role is electron donation and their further transfer, has been described and proven, i.e., nitrate and iron reductases, dependent on the nicotinamide adenine dinucleotide (NAD+)/NADH and the nicotinamide adenine dinucleotide phosphate NADP+/NADPH redox couples hydrogenase, and oxidase. Silver ions, due to their interaction with cytochromes and inhibition of electron transport, lead to disturbances in the functioning of the respiratory chain. The mechanism of silver nanoparticle synthesis in lactic acid bacteria was investigated in detail [351]. It was noted that the alkaline environment clearly favors the formation of nanoparticles as it catalyzes the enolization of monosaccharides. The resulting aldehyde is oxidized to carboxylic acid, while the metal ions are reduced to nanoparticles.

**Figure 8:**
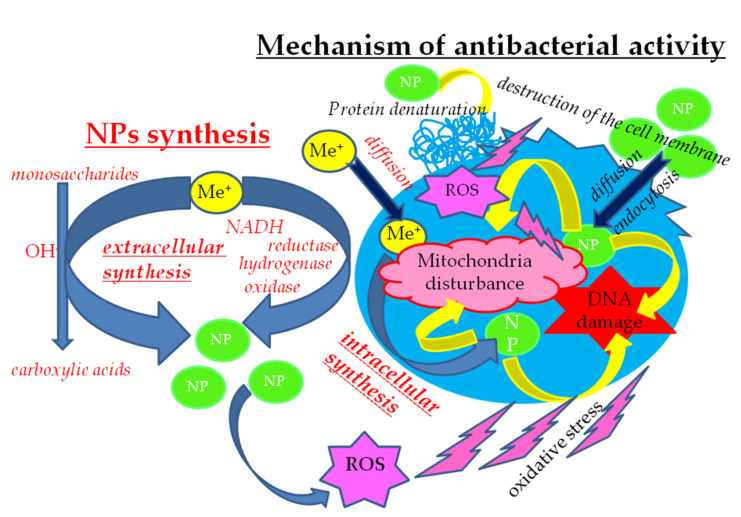
Schematic representation of intra- and extracellular NPs synthesis together with their possible mechanisms of antibacterial action.

The effect of nanoparticles on bacteria is complex and not fully understood due to the existence of numerous mechanisms of action (Figure 8). Bacteria and other microorganisms such as viruses, fungi, flagella, yeasts, and actinomycetes possess the ability to produce metallic NPs intracellularly as well as extracellularly. The studies showed the action of AgNPs on Escherichia coli and Staphylococcus ureus [352]. The appearance of pits in the bacterial envelope has been observed, which lead to a change in the electrostatic potential, an increase in the permeability of membranes, and damage to the DNA of the cell. Later, research was extended to other species of bacteria, which allowed for the emergence of two more mechanisms of action in the form of overproduction of free radicals (ROS) and the formation of complexes with various intracellular compounds, i.e., nucleic acids [353,354]. It is particularly interesting that even a short incubation with nanoparticles leads to the accumulation of chaperones and the S6 protein [355] and inhibition of the bacterial communication system (quorum sensing, QS), which is associated with a change in gene expression controlled by transcription regulators. It is known that disturbances in the functioning of these genes cause a change in the behavior of cells in the environment, e.g., the ability to create biofilms. This applies even to pathogenic bacterial strains such as Pseudomonas aeruginosa and Staphylococcus aureus. Biofilms create populations of microorganisms (bacteria, fungi, protozoa) that live at the interface [356]. They are surrounded by a protective substance composed of polysaccharides, proteins and nucleic acids, called a matrix. Biofilm formation is a multi-step process but is always initiated by adhesion. Within the biofilm, there may be synergistic or antagonistic interactions between the species inhabiting it, which may lead to the matrix disintegration [357]. The most dangerous are biofilms composed of pathogenic bacteria *E. faecalis*, *S. aureus*, Staphylococcus epidermidis, *E. coli*, *Klebsiella pneumoniae*, and *P. aeruginosa* [358], which are mostly responsible for hospital infections that are difficult to cure and are characterized by increased resistance to therapies [359]. The antibiotic resistance of biophimes is the result of, among other things, the presence of a matrix that is a barrier to drug penetration and the production of enzymes responsible for the hydrolysis of ß-lactam antibiotics [360,361]. Research on the potential of nanoparticles to combat biofilms meets the expectations of modern medicine. However, the number of publications on this topic has so far been rather small. It was shown that *P. aeruginosa* and *S. epidermidis* biofilms were inhibited in over 95% of cases by silver nanoparticles with a spherical shape and an average diameter of 50 nm [362]. The inhibition of biofilms formed by Multidrug-Resistant Klebsiella pneumoniae [363], Methicillin-Resistant Staphylococcus aureus [364], and Mycobacterium tuberculosis [365] has been proven. Unfortunately, the aggregation of nanoparticles inhibits their effective activity. Consequently, various stabilizers such as starch, citrate, and amino silica are used, and numerous composites composed of nanoparticles and other compounds have been identified. There are also reports on the inhibition of biofilm formation by AgNPs on medical devices, i.e., urological catheters, the surface of which was covered with nanosilver, were characterized by resistance to *E. coli*, *S. aureus*, and *Candida albicans*, even under continuous fluid flow conditions. [366]. Metallic/metal oxide nanoparticles, i.e., silver, gold, magnesium, titanium, zinc, aluminum, tantalum, and zirconium have been tested in orthopedics [367,368,369,370]. Nanoparticles embedded in implants and orthopedic scaffolds provide mechanical strength and antimicrobial protection. However, it should be remembered that many nanoparticles exhibit cytotoxicity and genotoxicity, especially in the case of their small size and higher concentrations [371].

Interesting examples of NPs that were produced by microorganisms are iron oxide nanoparticles which were produced by aquatic magnetotactic bacteria (MTB). These bacteria are able to biomineralize, magnetic magnetite, or greigite nanocrystallites called magnetosomes. When isolated from the MTB, magnetosomes exceed synthetic magnetic nanoparticles exhibiting promising anti-tumor efficacy against glioblastoma tumors in vivo tests [372,373]. It should be emphasized, however, that the anticancer activity is based on various mechanisms of action (heat, the release of chemotherapeutic drugs under a pH variation, nanoparticle excitation by radiation, and apoptotic tumor cell death). Magnetic nanoparticles are useful for targeted cancer therapies because they can be manipulated by external magnetic fields. Moreover, they are attracted toward hypoxic areas, such as the tumor regions, while retaining the therapeutic and imaging capacities of the isolated magnetosomes [374]. In nature, we can find other examples of a variety of nanomaterials synthesized by biological processes like example diatoms, which synthesize siliceous materials or S-layer bacteria forming NPs of gypsum and calcium carbonate layers.

### 5.2. Plant Extracts-Mediated NPs Synthesis

Plant extracts contain diverse compounds, which can be utilized as potent reducing agents, stabilizing agents, and precursor molecules for NPs formation [375,376]. In order to prepare the extracts, both the biomass of the whole plant and selected parts such as leaves, fruit, seeds, and above-ground parts can be used. The plant material can be fresh or powder-dried. Various techniques are used to prepare the extracts, but most consist of classical maceration with various solvents including water or water–alcohol mixtures. Nanoparticle synthesis is mediated by extract components with reducing potential including alkaloids, terpenoids, polyphenols, phenols, flavonoids, and proteins, which have additionally been identified as nanoparticle stabilizers. As compared with ordinary metal salts or initial materials alone, biologically synthesized nanoparticles have been found to be better scavengers of free radicals [377]. The antioxidant activity of NPs frequently depends on their size [378,379] as well as shape [380,381].

So far, many examples of the phytogenic synthesis of NPs have been described, including, among others, copper oxide and copper nanoparticles by the use of the leaf extract of Cissus arnotiana with antioxidant ability [382,383]. Apart from zinc oxide (ZnONPs), selenium (SeNPs), and nickel oxide nanoparticles (NiONPs), one of the biggest groups of plant-mediated NPs is iron nanoparticles (INPs). This group is divided into (a) iron oxide nanoparticles (IONs), (b) iron oxide hydroxide (FeOOH) nanoparticles, and (c) zero-valent iron (ZVI) nanoparticles [384,385,386,387]. Iron oxide (magnetite Fe_3_O_4_, magemite Fe_2_O_3_) NPs of certain sizes have superparamagnetic properties; therefore, they are useful as contrast agents and drug carriers.

The main problems encountered in the biogenic synthesis of nanoparticles concern achieving their appropriate shape, size, and monodispersity in the solution phase. Undoubtedly, the size and shape of NPs depend on the synthesis conditions and the chemical composition of the extract. Usually, optimization of synthesis conditions concerns such factors as the extract concentration, pH, temperature, and reaction or incubation time [388,389,390,391]. The reports on a plant-mediated approach to synthesize NPs by the use of different extracts are collected in Table 4.

### 5.3. Trends of NPs Modification

Increasing attention is paid to nanoparticles functionalized by various antioxidants obtained from various natural sources, such as algae, bacteria, fungi, lichens, and plants. It should be emphasized that most authors reported that the functionalized NPs exhibit a few times greater antiradical activity. The effective transport across the cell membrane through pinocytosis and the possibility of targeted localization give rise possibility of NPs utilization also as carriers for antioxidants. In those cases, inert metalcore and antioxidants attached to the nanoparticle surface can exert also independent activity [63]. In 2020, a review on antioxidant functionalized NPs was published [247]. Most papers present the synthesis of gold and silver nanoparticles that are easily functionalized with different small molecules of antioxidants, for instance, gold nanoparticles functionalized with tocopherol [440,441], gold nanoparticles coated with chitosan [442], silver NPs with glutathione [443], or more complex ones like graphite layered 30 nm cobalt nanomagnets with attached tocopherol derivatives [444]. Konopko et al. [440] and Nie et al. [441] prepared and characterized gold nanoparticles (AuNPs) coated with α-tocopherol-like residues. Both research groups proved that the assembly of chromanol groups on gold nanoparticles could efficiently enhance the activity of the vitamin E-derived antioxidant. In 2019, Mohd Taib et al. [445] synthesized Au-NPs utilizing water extract of *Hibiscus sabdariffa* leaves. Owing to the UV–VIS, FTIR, and HPLC analysis, chlorogenic acid was identified as the major antioxidant compound involved in the reduction of Au^3+^ ions. Moreover, the thiol groups can interact directly with the gold core to form gold–sulfur bonds (Au-S) responsible for the mucoadhesion properties of the synthetized AuNPs [446]. In 2017, Choi et al. [447] described nanoparticles modified by caffeic acid, which was immobilized on the surfaces of micro-dielectric barrier discharge (DBD) plasma-treated ZnO nanoparticles. Obtained nanoparticles showed strong antioxidant (ABTS), antibacterial activity against Gram-positive bacteria (*Staphylococcus aureus*), including resistant bacteria such as methicillin-resistant *S. aureus*, and against Gram-negative bacteria (*Escherichia coli*). Nanostructural materials such as nanotubes have been described as novel synergistic nano-antioxidants [448], for example, ascorbic acid loaded into the inner lumen of natural halloysite nanotubes [449] or halloysite externally deco-rated with tocopherol-like moieties and containing quercetin inside the nanotube [450]. Many studies have described functionalized silver and gold nanoparticles derived from fungal or bacterial extracts obtained from species *Ganoderma lucidium* [408,409,410], *Aspergillus versicolor*, *Cladosporium cladosporioides*, *Pestalotiopsis microspore* [401,403,451], and bacteria *Lactobacillus kimchicus* [419].

Another promising trend of nanobiotechnology represents the development of nano-drug delivery systems composed of biocompatible and biodegradable polymeric nanomaterials (polylactide-PLA, poly-lactic-co-glycolic acid- PLGA) that are able to encapsulate the therapeutic agent and progressively release it at the target site. Chlorogenic acid entrapped in hybrid materials composed of SiO_2_ and polyethylene glycol has been identified as a system able to control the overproduction of RNS/ROS [452]. Another example is curcumin encapsulated in a nanocarrier and covered with chitosan. Authors observed a protective effect of chitosan on the antioxidant activity of curcumin [453,454]. While inorganic nanoparticles, especially those with semiconductor properties, have found applications in in vitro diagnostics and imaging, nano-drugs ensure effective biodistribution thanks to the ability to overcome biological barriers. Thus far, many medicinal preparations in the form of nanoparticles have been developed, belonging to different classes of NPs (polymeric, inorganic, and lipid-based), such as polymer-drug conjugate, protein–drug conjugate, polymer–protein conjugate, antibody–drug conjugate, dendimeric drug, polymeric micelle, polymersome, liposome, PEGylated liposome, organic/inorganic colloid, quantum dot, Si-NPs, Au-NPs, and INPs. Extensive reviews on this subject have already been published [455,456,457,458]; unfortunately, it is beyond the scope of the current study.

Interesting nano-formulas are also doubly hydrophilic self-assembling block copolymers (DHBC), which in recent years have aroused more and more interest not only for the production of nanoparticles, but also as controlled drug distribution systems. A valuable review on DHBC was published in 2020 by Jundi et al. [459].

## 6. Concluding Remarks and Future Perspectives

Considering the key role of antioxidants to treat oxidative diseases, the development of reliable antioxidant activity assays of different products with high antioxidant content, as potential drugs or supplements, is needed. Several analytical techniques can be applied for this purpose such as spectroscopic, chromatographic, and electrochemical ones. At the beginning of the 21st century, antioxidant assays based on NPs were developed. The use of NPs as optical or electrochemical probes appears to be a very promising approach; however, this technique has still been scarcely followed. Over 5 years of research on the NPs-based method has resulted in a negligible number of publications, which illustrates the fact that in the PubMed database, the phrases “antioxidant capacity”, “nanoparticles”, and “plant extracts” are associated with no more than 70 scientific papers. One should emphasize that performing the comparative analysis of antioxidant potentials on the basis of results published by different research groups is very difficult. The antioxidant potential of natural products, and even single chemicals, depends on many factors such as conditions of samples collections, as well as the extracts preparation method and the way of expressing results.

On the other hand, the plant extracts rich in antioxidants that act as both reducing and stabilizing agents appear to be useful for creating metallic nanoparticles. Green synthesis surpasses classical methods, providing such benefits as low-cost, environmentally friendly strategies not requiring high pressure, energy, temperature, or external toxic chemical agents. Furthermore, green synthesis ensures the formation of nanoparticles free of toxic contaminants, which makes them suitable in therapeutic applications such as antimicrobial agents in bandages, applications in targeted drug delivery, or clinical diagnostics as contrast agents (MRI-Magnetic Resonance Imaging). The popularity of green nanoparticle synthesis toward bio and medical applications is reflected in the number of around 5000 publications that have appeared in the PubMed database in the last five years.

A promising trend that has been developing dynamically in recent years is the synthesis of antioxidant functionalized nanoparticles. Such modification improves the bioavailability of antioxidants providing the benefits of biocompatibility, high stability, and targeted delivery.

## Figures and Tables

**Table 1:** Examples of reactive species. Reproduced with permission from Graves, D.B., [*J. Phys. D Appl. Phys.*]; published by IOP Publishing, 2012 [47].

Reactive Species	Form	Example
Reactive oxygen species (ROS)	Radical	HO^•^, ^1^[O]_2_, O_2_^•−^ HOO^•^, ROO^•^, RO^•^, CO_2_^•−^, CO_3_^•−^
	Non-radical	O_3_, H_2_O_2_, HOCl, HOI, HOBr, ROOH, CO, ONOOH, ONOO^−^, O_2_NOO^−^, HOOCO_2_^−^, (O_2_ 1Dg)
Reactive nitrogen species (RNS)	Radical	NO^•^, NO_2_^•^, NO_3_^•^
	Non-radical	ROONO, RO_2_ONO, CH_3_C(O)OONO_2_, N_2_O_4_, N_2_O_3_, N_2_O_5_, HNO_2_ NO_2_Cl NO^−^, NO^+^
Reactive chlorine species	Radical	Cl^•^
	Non-radical	ClBr, Cl_2_, ClO_2_
Reactive sulfur species	Radical	S^•^
	Non-radical	H_2_S, RSSR, RS(O)SR, RSOH, RS(O)_2_SR, RSR’

**Table 2:** Examples of the non-enzymatic assays used for in vitro determination of antioxidant capacity with distinguished chromogenic agents, observed changes, the principle, mode, and mechanism of the assay (Mech).

Assay	The Chromogenic Agents	Observed Changes	Principle of Assay	Mode	Mech	Ref
Total antioxidant capacities						
Crocin bleaching	crocin	bleaching of crocin	The ability of AOs to inhibit oxidation of crocin.	Abs. 443 nm pH = 7.0–7.5	HAT	[125,126]
ORAC (Oxygen radical absorbance capacity)	fluorescein, dichloro- fluorescein	fluorescence decay	The fluorescence caused by oxidation of the probe by peroxyl-radical initiated by thermal decomposition of AAPH, is delayed/inhibited by AOs.	Fl. λ_ex_ = 485 nm λ_em_ = 538 nm pH = 7.4	HAT	[127]
TRAP (Total peroxyl radical trapping antioxidant parameter)	β-phycoerythrin	fluorescence decay	Fluorescence decay along time due to oxidation of the probe is delayed by AOs.	Fl. λ_ex_ = 495 nm λ_em_ = 575 nm pH = 7.5	HAT	[128,129]
β-carotene bleaching assay	β-carotene	bleaching yellow color of β-carotene	The ability of AOs to slow down the rate of β-carotene bleaching due to its reaction with peroxyl radicals, which are formed by linolenic acid oxidation.	Abs. 470 nm pH = 5.5–7.5	HAT	[130,131]
PCL (Photochemiluminescence)	luminol	blue light emision	An AO-sensitive inhibition of a photo-induced, chemiluminescence accompanying autooxidation of luminol.	Cl. 360 nm pH = 10.5	HAT	[132,133,134]
Reducing antioxidant power (RP)						
FRAP (Ferric reducing antioxidant potential)	ferric tripyridyl triazine	yellow color to blue	AOs as reductant at low pH can reduce ferric tripyridyl triazine to ferrous form, causing absorbance increase.	Abs. 593 nm pH = 3.6	ET	[135]
CUPRAC (cupric ion reducing antioxidant capacity)	Cu(II) complex	light blue to orange-yellow	Ability of AO for the reduction of Cu(II) in bathocuproine(2,9-dimethyl-4,7-diphenyl-1,10-phenanthroline) or neocuproine (2,9-dimethyl-1,10-phenanthroline) complexes to Cu(I) forms.	Abs. 490 nm 450 nm pH = 7	ET	[136]
CERAC (Ce(IV)-based reducing capacity)	Ce (IV)	fluorescence	The ability of AO to reduce Ce(IV) to Ce(III) accopanied with fluorescence elevation.	Fl. λ_ex_ = 256 nm λ_em_ = 360 nm pH acidic	ET	[137,138]
CHROMAC (Chromium reducing antioxidant capacity)	Cr (VI) with DPC	red–violet product	The reduction of chromate(VI) to Cr(III) in acidic solution. The remaining Cr(VI) reacts with DPC to produce a chelate complex. The Cr(VI) consumption was correlated with AO’ concentration.	Abs. 540 nm. pH = 2.8	ET	[139]
Phosphomolybdenum assay	Phosphormolybdenum complex	green product	The reduction of Mo(Vl) to Mo(V) by AO.	Abs. 695 nm pH acidic	ET	[140]
The Folin–Ciocalteu (FC) assay	Tungstate–molybdate complexes	from yellow to dark blue	FC reagent in a basic medium is able to oxidize reducing substances, mainly phenolic and polyphenolic AOs. The change in color is connected with transformation of Mo(VI) to Mo(V), causing absorbance increase.	Abs. 750–765 nm pH = 10	ET	[109]
PFRAP (Potassium ferricyanide reducing power assay)	Ferricyanide reagent: Fe(III), Fe(CN)_6_^3−^	prussian blue	The AOs react with potassium ferricyanide Fe(CN)_6_^3−^) forming potassium ferrocyanide Fe(CN)_6_^4−^ which further reacts with FeCl_3_ to form prussian blue KFe[Fe(CN)_6_].	Abs. 700 nm pH = 6.6	ET	[141]
FTC (Ferric thiocyanate)	Fe(S-CN)_2_	red color	A hydroperoxide formed from a lipid (linoleic acid) oxidizes a ferrous ion to a ferric ion. The AO causes an inhibitory effect on hydroperoxide formation or by its ability to donate an electron to ferric ion.	Abs. 500 nm	ET	[142,143]
FOX (Ferrous Oxidation-Xylenol Orange Assay)	ferric-XO complex	blue-purple color	The presence of hydroperoxides that oxidize ferrous ion to ferric ion, which subsequently react with xylenol orange (XO).	Abs. 550 nm.	ET	[144]
Assays associated with lipid peroxidations						
LPO (Lipid peroxidation inhibition assay)	N-methyl-2-phenylindole	dye product	AOs delay radical-induced malonyl dialdehyde generation. MDA and HAE are measured as an indicator of lipid peroxidation. The product -MDA with chromogenic reagent gives carbocyanine adduct.	Abs. 586 nm	ET	[138,145]
TBARS (Thiobarbituric acid reactive substances assay)	TBARS	red-pink color	The reaction of lipid peroxidation products (MDA), with TBA, leads to the formation of MDA-TBA adducts (TBARS).	Abs. 532 nm pH = 4	ET	[130,131,146,147]
Conjugated diene assay	linoleic acid	UV absorbance	Antioxidants delay conjugated dienes formation. The AO effect can be evaluated by monitoring the conjugated diene formation.	Abs. 234 nm		[148]
Radical scavenging assays						
DPPH	2,2-diphenyl-1-picrylhydrazyl radical	deep violet to pale yellow or colorless	The decrease in DPPH absorbance depends linearly on AO’ concentration.	Abs. 515–517 nm pH = 7	HAT/ET	[130]
ABTS	2,2’-azino-bis(3-ethylbenzothiazoline-6-sulfonic acid (ABTS^+.^)	bluish-green to colorless	ABTS treated with Na/K persulphate or MnO_2_ gives a radical cation (ABTS^+^). ABTS^+.^ is reduced by antioxidants. The decrease in absorbance depends linearly on AO’concentration.	Abs. 734 nm pH = 7.4	HAT/ET	[149]
DMPD (N,N-dimethyl-p-phenylene-diamine)	DMPD^·+^ radical cation	reduction of purple color	DMPD^·+^ is generated through a reaction between DMPD and potassium persulphate the assay measures scavenging of free radicals by AOs.	Abs. 517 nm pH = 5.25	HAT	[150,151]
SOSA (Superoxide Anion Radical Scavenging Capacity)	NBT	yellow to blue	The ability of the AO to compete with NBT to scavenge O_2_^•−^ generated by an enzymatic HPX-XOD, X-XOD or PMS/NADH systems.	Abs. 560 nm pH = 7.4	ET	[135,152]
Nitric oxide free radical scavenging activity	Griess reagent	colorless to light pink to deep purple	NO was generated from sodium nitroprusside and measured by the Greiss reaction. AO reduces the amount of nitrite.	Abs. 546 nm pH = 7.4	ET	[153]
Peroxynitrite Scavenging Capacity Assay	Evans Blue	dye bleaching	The percentage of scavenging of ONOO^−^ by the Evans Blue was measured in presence of AO.	Abs. 611 nm pH < 7	ET	[154]
HORAC (Hydroxyl Radical Averting Capacity Assay)	fluorescein	fluorescence decay	OH radicals are generated by a Co(II)-mediated Fenton-like reaction. The reaction is confirmed by the hydroxylation of p-hydroxybenzoic acid. Metal ion-induced OH radical generation reaction can be monitored by the fluorescence decay of fluorescein. In the presence of AO, the formation of OH radicals can be inhibited because the metal is deactivated due to coordination with AO.	Fl. λ_ex_ = 493 λ_em_ = 515 nm	HAT	[130,135,146,147,148,149,150,151,152,153,154,155]
HRS (Deoxyribose Degradation Assay)	MDA-TBA adducts	pink	A mixture of Fe(III)-EDTA, H_2_O_2_, vit.C generates OH radical, is able to degrade deoxyribose. The products heated under acidic conditions form MDA detected by adduct with TBA. AO can inhibit deoxyribose damage.	Abs. 532 nm pH = 7.4	ET	[156]
Hydroxyl Radical Scavenging Capacity Assay	Fenton-like system Fe(II)^/^H_2_O_2_	-	The Fenton system generates a constant flux of pure OH radicals. ESR measurements evaluate the OH radicals scavenging capacity of AOs.	Electron spin resonance (ESR)	ET	[157]
CAA(Cellular Antioxidant Activity Assays)	DCFH-DA	fluorescence decay	The ability of AOs to prevent oxidation of DCFH by azide generated peroxyl radicals in human hepatocarcinoma HepG2 cells.	Fl. λ_exc._502 nm, λ_em_ 520 nm	ET	[158]
Nonradical reactive oxygen species scavenging assay						
Hydrogen peroxide scavenging activity	hydrogen peroxide	UV absorbance	Hydroxyl radicals are the byproducts of H_2_O_2_ decomposition. They initiate lipid peroxidation. After the addition of AO, the absorbance is measured against blank (phosphate buffer).	Abs. 230 m pH = 7.4	ET	[159]
Singlet oxygen scavenger	RNO	bleaching of RNO	Production of singlet oxygen (1O_2_) was achieved by monitoring RNO bleaching. Singlet oxygen was generated by a reaction between NaOCl and H_2_O_2_.	Abs. 440 pH = 7.1	ET	[160]
ACA (Aldehyde/carboxylic acid assay)	Alkylaldehyde/ alkylcarboxylic acid	-	The stoichiometric conversion from alkylaldehyde (hexanal) to alkylcarboxylic acid in the presence of radicals induced by heat, O_2_, or H_2_O_2_.	GC	ET	[161]
			Metal chelating capacity assays (MCA)			
Ferrous ions chelating assay	Fe(II) with 2,2-bipyridine or ferrozine	blue	The capacity to chelate ferrous ion can be disturbed by the presence of other complexing agents (AOs), which cause a decrease intensity of the complex (Fe(II) and ferrozine).	Abs. 562 nm 522 nm pH = 4–10	ET	[162]
Copper(II) chelating capacity assay	Cu(II)- PV	dark to yellow	The chelating activity can be estimated by the measurement of the rate of color reduction.	Abs. 632 nm pH = 6	ET	[163]
Nanoparticles (NPs)-based assays						
Gold nanoparticles (Au-NPs)	NPs	No color into dark red	The highest capacity of reducing gold(III) to gold NPs corresponds to the highest antioxidant activity. Alternatively, cyclic voltammetry measures anodic peak potentials	Abs. 555 nm pH = 8	ET	[117,118]
Silver nanoparticles (Ag-NPs)	NPs	no color into pale yellow	Nanoparticles generated from metal salts upon reduction with antioxidants in the presence of citrate-stabilized silver seeds.	Abs. 423 nm pH = 7	ET	[118]

Abbreviations: AAPH (2,2′-azobis–2–methyl-propanimidamide,dihydrochloride); Abs. (Absorbance); DPC (1,5-diphenylcarbazide); XO (xylenol orange); MDA (malondialdehyde); HAE (4-hydroxyalkenals); NBT (nitroblue tetrazolium); Griess reagent (1% sulfanilamide, 2% H_3_PO_4_, and 0.1% naphthylethylenediamine dihydrochloride); DCFH (dichlorofluorescein); Luminol (5-amino-2,3-dihydrophthalazine-1,4-dione); Cl. (Chemiluminescence), Abs. (Absorbance); Fl. (Fluorescence); HPX-XOD (hypoxanthine–xanthine oxidase); X-XOD (xanthine–xanthine oxidase); PMS/NADH (phenazine methosulphate systems); AO (antioxidant); PV (pyrocatechol violet); RNO (N, N-dimethyl-p-nitrosoaniline); DCFH-DA (2’,7’-dichloro-dihydrofluorescein diacetate); triazine (2,3,5- triphenyl-1,3,4-triaza-azoniacyclopenta-1,4-diene chloride).

**Table 3:** Examples of antioxidant capacity assessment of extracts obtained from different species.

Analysed Product	Antioxidant Assays	Positive Control	Extraction Procedure	Ref.
Plants				
*Bryonia alba* L.	DPPH, CUPRAC, FRAP, TEAC, SNPAC	quercetine, BHT, Trolox	Fifty grams of powder was macerated with 500 mL methanol for 24 h. After percolation, extract was evaporated under vacuum at 40 °C.	[279]
*Cistus ladanifer* L., *Cistus salvifolius* L., *Cistus albidus* L., *Erica australis* L., *Arbutus unedo* L., *Pistacia lentiscus* L.	DPPH, FRAP, ABTS, RP	quercetin	Twenty grams of grounded leaves was mixed with 200 mL of methanol. The mixture was kept for 24 h at RT. Then, it was filtered.	[280]
*Prunus avium*, *Prunus persica*, *Prunus domestica*, *Olea europeae*, *Pirus communis*, *Pirus maus*, *Pistacia verra*, *Castanea sativa*	DPPH, FRAP, TAC	Trolox, ascorbic acid	Two grams of sample was mixed with 60% methanol and kept 1 h at dark at RT. The procedure was repeated two times. The combined extracts were centrifuged and filled to 50 mL by aqueous methanol.	[281]
*Vitis vinifera* L. (*Maraština*, *Pošip*; *Lasin*, *Merlot*, *Syrah*, *Vranac*)	DPPH, FRAP	Trolox	The dry plant material (20 g) was extracted using 100 mL of ethanol/water 80/20, (*v*/*v*) at 60 °C, for 60 min. The extract was filtered and dried under a vacuum at 50 °C. The dry residues were redissolved with 50% methanol-water and centrifuged at 5000 rpm for 10 min.	[282]
*Ornithogalum billardieri*	β-carotene-linoleic acid assay, ABTS, MCA/ferrous ion, TAC/H_2_SO_4_, Na_3_PO_4_, (NH_4_)_2_MoO_4_)	ascorbic acid	The combined maceration with sonication either at 25 °C for 50% ultra-sound (US) treatment or the extract obtained under the optimal conditions: extraction time: 37.1 min, temperature: 44.2 °C, water volume-to-mass ratio: 33.8 mL/g, and US%:51.7%	[283]
Apple cultivars: antonówka, delikates, Early Geneva, papierówka, Paulared, Sunrise, Quinte Gloster, Jonagored, Ligol, Rubinola	DPPH, FRAP, MCA/ferrous ion	Trolox, catechin	The material was lyophilized. A total of 250 mg of sample was extracted by sonication using 1 mL of 80% methanol for 30 s. Then, the mixture was vortexed, centrifuged for 5 min (13,200 rpm), and sonicated. The extraction procedure was repeated five times. The supernatants were collected together.	[209]
*Fraxinus angustifolia* Vahl	TAC/Folin-Ciocalteu, FRAP, ABTS, DPPH	α-tocopherol	Manna samples (10 g) were dissolved in methanol-water (2:1) and extraction carried out at 25 ± 2 °C and in darkness for 1 h., and centrifuged (3000 *g*, 10 min). The supernatants were filtered and evaporated at 35 °C. Dried samples were resuspended in 5 mM phosphate buffer saline pH 7.4.	[284]
*Chenopodium quinoa*	DPPH, FRAP	Trolox, gallic acid	Dry plant materials (roots, leaves, stems, flowers, and seeds) were extracted by the use of ultrasound-assisted extraction with 96% ethanol. The extracts were concentrated under reduced pressure in a rotary evaporator.	[239]
*Lycium barbarum*, *Lyciumchinense*	DPPH, ABTS	-	Samples were dried, powdered, and dissolved in distilled water.	[285]
*Aegopodium podagraria* L.	DPPH	GSH, ascorbic acid	A total of 2.5 g of air-dried or fresh aerial parts was extracted by 100 mL of 80% (*v*/*v*) ethanol. The samples were kept at RT for 3 days, three months in dark, or in an ultrasonic bath for 60 min. Then, the extracts were filtered.	[286]
Fungi				
*Achaetomium* sp.	DPPH	ascorbic acid, BHT, gallic acid, pyrogallol	The organic ethyl acetate extract was evaporated. The crude extract was dissolved in DMSO.	[287]
*Acremonium charticola*, *Rhizopus oryzae*	ABTS	ascorbic acid	Fungi were cultured in potato dextrose broth at 37 °C. After 3 days, the cultures were centrifugated at 5000 rpm for 10 min. The filtrate (1 g) was mixed with 100 mL methanol and ultrasonicated for 30 min. The homogenate was created for three days at RT. Then, it was evaporated with a rotary vacuum evaporator (50 °C, 100 rpm) to the volume of 25 mL.	[288]
*Agaricus bisporus*, *Pleurotus ostreatus*, *Pleurotus eryngii*, *Lentinula edodes*	TPC/Folin–Ciocalteu, Ferricyanide/prussian blue, DPPH, TBARS β-carotene/linoleate	Trolox, gallic acid	The product was lyophilized. The obtained powder was mixed with methanol and kept at 25 °C at 150 rpm for 1 h. Then, the mixture was and filtered. The extracts were evaporated under reduced pressure and redissolved in methanol at a concentration of 20 mg/mL.	[171]
*Aspergillus wentii*, *A. wentii*, *Penicillium citrinum*, *Penicillium granulatum*	DPPH, FRAP, MCA/ferrous ion, NO^•^ scavenging activity, RP/potassium ferricyanide	ascorbic acid, BHT, rutin, catechin	The fungal mycelia were grown on agar plates with extracts of yeast and glucose. After 6 to 7 days, the Czapek–Dox’s broth was inoculated of fungal mycelia. After 10 days of incubation at 25 °C, the culture broth was filtered.	[289]
*Aspergillus niger*, *Aspergillus peyronelii*	DPPH, RP H_2_O_2_ scavenging activity	ascorbic acid	The dried samples were extracted by the use of ethyl acetate (1:10) applying cold percolation for 48–72 h. Then, obtained extracts were filtered, and concentrated under vacuum at 40 °C.	[290]
*Aspergillus versicolor*	ABTS, DPPH	Trolox	Aspergillus versicolor was cultivated on rice for 30 days. The EtOAc extracts of solid fermentation were fractioned through slica gel and Sephadex LH-20 column chromatography (CC), and were further purified by semi-preparative HPLC.	[291]
*Auricularia auricular*	ABTS, O_2_^●−^, OH^•^ scavenging activity, lipid peroxidation	-	*A.auricula* was extracted by hot water and ultrasonic-assisted extraction. The supernatants were precipitated with absolute ethanol (95%) and maintained at 4 °C overnight. The precipitate was centrifugated, dissolved in DI, and dialyzed. The non-dialyzed portion was lyophilized to give a crude polysaccharide extract. The separation of polysaccharides was performed using CTAB or CPC.	[292]
*Cephalosporium* sp.	DPPH	gallic acid	The fermented material (1.75 kg) was extracted twice with EtOAc (9.0 L) for 3 days at RT, and the extract was evaporated under vacuum.	[293]
*Cerrena unicolor*	ABTS, DPPH	Trolox, ascorbic acid	Ten-day-old cultures were filtered and washed with DW. The fungal biomass was used for the polysaccharides extraction by hot water (90 °C, 4 h). The proteins were separated by anion exchange chromatography on a DEAE Sepharose column with a linear gradient of NaCl (0.1–0.5 M). The culture liquid was subdivided into two fractions by ultrafiltration: substances above 10 kDa (rude lactase) and substances below 10 kDa with low molecular weight metabolites.	[294]
*Flammulina velutipes*, *Hypsizygus tessellatus*	DPPH, H_2_O_2_ scavenging activity, FRAP	gallic acid, quercetin	The stems of the mushrooms were dried at 60 °C and pulverized. Samples (180 g) were extracted overnight with 500 mL of water, absolute methanol, 95% acetone, or 95% ethyl acetate at RT. The extracts were filtered, evaporated to dryness. Aqueous fractions were concentrated to 50 mL, freeze-dried, and stored at 4 °C.	[295]
*Grifola frondosa*	DPPH, β-carotene bleaching assay, inhibition of lipid peroxidation, RP, CUPRAC	quercetin	Mushrooms were boiled in DW at the ratio of 1:10 (*w*/*v*) for 30 min. Then, the extract was filtered, and freeze-dried.	[296]
*Penicillium expansum*	DPPH, RP, FRAP MCA/ferrous ion, NO^•^ scavenging activity	-	The fungi were grown on CDM, MEM, PDM, and YEM. After incubation at 25 °C for 10 days, the culture broth was filtered through filter paper. The culture broth was extracted with petroleum ether, chloroform, ethyl acetate, and butanol. Then, the extracts were then evaporated to dryness in a vacuum, and the precipitates were dissolved in DMSO.	[297]
*Phlebia brevispora*, *Phlebia floridensis*, *Phlebia radiate*, *Phlebia fascicularia*	DPPH, FRAP, RP, MCA, NO^•^ scavenging activity	-	Five grams of dried wheat straw was ground, washed, and dried at 90 °C. Next, it was moistened by the use of 25 mL of malt extract (0.5%, *w*/*v*) and inoculated with three mycelial discs (8 mm), which were grown on YGA plates for 6 days. The inoculated flasks were kept at 25 °C for 30 days. Then, they were homogenized, filtered, and dried at 90 °C.	[298]
*Pleurotus florida*, *Pleurotus sajor-caju*, *Pleurotus cystidiosus*, *Pleurotus djamor*	FRAP, DPPH, RP, MCA, H_2_O_2_, O_2_^●−^ scavenging activity	-	Sporophores were cleaned and dried at 40 ± 2 °C for 10–12 h. The obtained powders (100 g) were refluxing with light petrol (60–80 °C) for 6 h with the aim of defatting. Then, the material was extracted with 95% ethanol (500 mL × 3) by refluxing for 6 h. The extracts were combined, filtered, and evaporated to dryness at 40 °C. The dried extracts were redissolved in methanol at a concentration of 20 mg/mL for analysis.	[299]
*Trametes versicolor*, *Trametes hirsuta*, *Trametes gibbosa*	ABTS, FRAP	ascorbic acid	Dried material (3.0 g) was grounded. Extraction was carried out in 96% ethanol during 72 h. The extracts were centrifuged and supernatants filtrated. The filtrates were concentrated under reduced pressure at 40 °C to dryness and redissolved in 96% ethanol	[300]
Bacteria				
*Bacillus coagulans* RK-02	β-carotene-linoleate, O_2_^●−^, OH^•^ scavenging activity, DPPH	ascorbic acid, α-tocopherol	The 36 h culture was centrifuged at 10,000× *g* for 20 min at RT. The supernatant was filtered. The proteins were isolated by the addition of 10% TCA. After 12 h at 4 °C, the mixture was centrifugated. Four volumes of 95% ethanol were added to the supernatant and centrifuged. The pellet was lyophilized, dissolved in 5 mL DW and dialyzed (MWCO 12,000 Da).	[301]
*Weissella cibaria* GA44	DPPH, RP, O_2_^●−^, OH^•^ scavenging activity	ascorbic acid	The cultures were heated at 100 °C for 10 min. The cells were removed by centrifugation at 12,000× *g* for 15 min at 4 °C. The supernatant was precipitated with double volume of chilled ethanol, shaken, and centrifuged at 98 5000× *g* for 30 min at 4 °C. The precipitate was dried at 50 °C, and dissolved in water. This step was repeated three times and dialyzed against distilled water for two days at 4 °C using 10 kDa dialysis membrane and then lyophilized.	[302]
*Lactobacillus plantarum* C88	OH^•^ scavenging activity, DPPH, the LPC-1 on H_2_O_2_^−^ induced oxidative stress in Caco-2 cells	ascorbic acid	After 20 h of the incubation period, TCA was added to achieve 4% (*w*/*v*). The mixture was stirred for 30 min at RT. Cells were removed by centrifugation (10,000× g, 4 °C, 15 min). Crude EPS was precipitated by the addition of 2 volumes of cold ethanol. Crude EPS was collected by centrifugation. The pellet was dissolved in deionized water and dialyzed (MW cut-off 3500 Da) for 24 h against distilled water at 4 °C and then lyophilized.	[303]
*Pseudomonas hibiscicola*, *Macrococcus caseolyticus*, *Enterobacter ludwigii*, *Bacillus anthracis*	DPPH	ascorbic acid	The bacteria were cultured in 500 mL broth at 35 °C. Then, the culture was centrifuged at 8000× *g* for 5 min. The supernatant was extracted with ethyl acetate (ratio of 1:1). Then, the extract was concentrated to dryness in a rotatory evaporator at 37 °C. The solids were re-dissolved in 20% DMSO and filtered.	[304]
*Alteromonas* sp. *Shewanella* sp. *Serratia* sp., *Citricoccus* sp., *Cellulophaga* sp., *Ruegeria* sp. *Staphylococcus* sp.	DPPH, ORAC	BHT, Trolox	Isolated bacteria were cultured in 500 mL of Marine Broth for 3 days at 25 °C. Bacteria cells were centrifuged, and the pellet was lyophilized. Then, the lyophilizes were extracted with methanol and dichloromethane (1:1) for 12 h. The solvents were evaporated at 40 °C, and the extracts were re-dissolved in DMSO.	[305]
*Pseudomonas* sp. (HR04)	anti-lipoperoxidative activity	BHT, α-tocopherol	The mycelial cake was extracted with acetone followed by purification by column chromatography on silica gel and Sephadex LH-20.	[306]
*Lactobacillus casei* CRL 431 (IC431)	LPO, ABTS, ORAC, CAT, GPx	Trolox	An aliquot (10 mL) of bacteria suspended in PBS was mixed with lysozyme (1 mg/mL) and incubated at 37 °C for 150 min. Then, cells were disrupted by sonication in an ultrasonic processor at 10 °C. After centrifugation (3600× *g*, 4 °C, 10 min), the supernatant was collected and stored in dark at 4 °C.	[307]
*Lactobacillus fermentum*	DPPH, ABTS, FRAP, OH^•^ scavenging activity	ascorbic acid	The cells were washed with 0.85% NaCl and sonicated. The obtained fluid was mixed with 75% ethanol. The precipitate was collected, redissolved, deproteinized, purified on an anion exchange column eluting with deionized water, 0.1 M, and 0.3 M NaCl and subsequently loaded onto a Sephadex G-100 column and eluted with DW. The collected fractions were lyophilized.	[308]
*Lactobacillus plantarum* (LAU103)	ABTS, DPPH, ORAC, MCA/ferrous ion, OH^•^ scavenging activity	ascorbic acid	A total of 5 mL of crude EPS solution (20 mg/mL) was separated with DEAE-cellulose column using deionized water, 0.2 and 0.5 M NaCl as eluent. Peak fractions containing polysaccharides were pooled, dialyzed, and lyophilized. Then, the fraction was further purified on a Sepharose CL-6B gel column and eluted with 0.9 M NaCl solution.	[242]
*Lactobacillus paracasei* *subsp. paracasei* NTU 101 (101EP), *Lactobacillus plantarum* NTU 102 (102EP)	DPPH, MCA/ferrous ion, inhibition of linoleic acid peroxidation, RP	ascorbic acid	The cultures were centrifugated at 5000× *g* at 4 °C for 15 min. The supernatant was added to 0.4 TCA at 4 °C for 3 h. Then, the supernatant was mixed with ethanol at 4 °C for 24 h, followed by centrifugation. The obtained precipitate was dialyzed for 24 h and lyophilized.	[309]
Algae				
Astaxanthin	FRAP, TEAC ORAC, DPPH	tert-Butyl alcohol	Astaxanthin was dissolved in tetrahydrofuran (THF).	[244]
*Desmarestia antarctica*, *Iridaea cordata*	RP, TPC, DPPH	gallic acid, ascorbic acid	The samples were rinsed with Milli-Q water, cut into fine pieces, then boiled at reflux for 15 min. The flask was moved to an ice bath to complete the extraction. The extract was thus centrifuged at 4500 rpm for 10 min, and the supernatant was filtered and stored at 4 °C.	[310]
*Haematococcus pluvialis*, synthetic astaxanthin	ABTS, ORAC, CAA	Trolox	The extracts were obtained by solvent using DMSO or supercritical extraction (AstaCO_2_).	[311]
astaxanthin	ORAC-EPR	-	The acetonitrile solutions of Catechin, epicatechin, epigallocatechin gallate, kaempferol, myricetin, resveratrol, and astaxanthin were diluted with phosphate buffer containing DM-β-CD.	[312]
*Dunaliella salina*, *Tetraselmis chuii*, *Isochrysis galbana* *clone* Tahiti.	DPPH	-	Methanolic extracts were prepared in different concentrations (50, 100, 250, 500, and 1000 ppm).	[313]
*Galderia sulphuraria*, *Neochloris texensis*, *Stichococcus bacillaris*, *Ettlia carotinosa*, *Chlorella minutissima*, *Schizochytrium limacinum*, *Crypthecodinium cohnii*, *Chlorella vulgaris*	DPPH	-	Methanol extraction: 20 mL methanol was added to 0.5 g dry biomass and sonicated (9 cycles, 50% power) for 20 min. Then, samples were centrifuged at 3500 rpm for 5 min. Pellets were re-extracted in 20 mL methanol 3 times and the supernatants were collected. The samples were filtered and evaporated at 40 °C. Hot water extraction: 1 g of dry sample was added to 100 mL DW and boiled for 30 min. After cooling, extracts were centrifuged at 3500 rpm for 10 min, and supernatants were freeze-dried.	[314]
*Chlorella vulgaris* *Spirulina platensis*	ORAC, DPPH, FRAP	Trolox	The extracts were obtained by the use of ultrasound-assisted extraction by water/ethanol (50:50, *v*/*v*).	[315]
*Scenedesmus subspicatus*	DPPH	catequin, gallic acid	Different solvents such as ethanol, methanol, butanol, acetone, DMSO, and water were used for extraction. One gram of dried samples were mixed with 10 mL for each solvent. The extraction was carried out for 30 min by sonication (40 kHz) in an ultrasonic bath followed by a 2 h shake, and centrifugation for 10 min.	[316]
*Tetraselmis suecica*	DPPH	α-tocopherol	A total of 100 mg of freeze-dried biomass was extracted with 1 mL ethanol/water (3:1, *v*/*v*) for 30 min. The mixture was centrifuged at 4500× *g*, for 10 min, at 20 °C. Then, the ethanolic phase was dried.	[317]
Lichens				
*Cetraria islandica* (L) Ach.	DPPH, the thiocyanate method, RP, O_2_^●−^ scavenging activity	α-tocopherol, BHT, BHA	For water extraction, 20 g sample was mixed with 400 mL boiled DW and stirred for 15 min. Then, the extract was filtered. The obtained filtrates were frozen and lyophilized.	[261]
*Usnea ghattensis*	ABTS, O_2_^●−^ scavenging activity, lipid peroxidation/linoleic acid	Trolox BHT, BHA, quercetin	Cell mass (14.8 g dry wt) was extracted using 20 mL of 10% (*v*/*v*) acetone, dimethyl sulphoxide (DMSO), methanol or light petroleum (40–60 °C) at RT. The extracts were then filtered, concentrated 4-fold under vacuum, and freeze-dried and then dissolved in 1 mL of acetone, DMSO, methanol, or water for the preparation of test stock solutions.	[262]
*Parmelia saxatilis*, *Platismatia glauca*, *Ramalina pollinaria*, *Ramalina polymorpha* *Umbilicaria nylanderiana*	DPPH, the inhibition of linoleic acid oxidation	gallic acid	Air-dried and powdered lichens (10 g) were mixed with 250 mL of methanol. The extraction was conducted in the Soxhlet apparatus for 72 h at a temperature of the boiling point of the solvent. The extracts were filtered and then concentrated in vacuo at 40 °C.	[263]
*Anaptychya ciliaris*, *Nephroma parile*, *Ochrolechia tartarea* *Parmelia centrifuga*	MCA/ferrous ion, TPC/Folin-Ciocalteu reagent, RP	Trolox, ascorbic acid	One hundred grams of pulverized dried lichen were extracted with 1 L of methanol using a Soxhlet apparatus for 72 h. The obtained extracts were filtered and then concentrated under reduced pressure.	[266]
*Parmotrema praesorediosum*, *P. rampoddense*, *P. tinctorum* *P. reticulatum*	DPPH	-	Powdered lichen samples (250 g) were subjected to soxhlet extraction using acetone and methanol. The extracts were then filtered through filter paper, concentrated in vacuo, and air-dried.	[268]
Actinomycetes				
*Streptomyces* (R56-07)	DPPH	α-tocopherol	The ethyl acetate extract of the fermentation broth was subjected to silica gel MPLC and for further purification to a Sephadex LH-20 column and RP-HPLC.	[271]
*Streptomyces chromofuscus*	the inhibition of lipid peroxidation, DPPH	α-tocopherol BHT	Carbazole compounds, carazostatin, carbazomycin B and their chemically modified derivatives were isolated from the culture of *Streptomyces chromofuscus* by the use of chromatography on silica gel with hexane-EtOAc (20:1) as an eluent.	[318]
*Streptomyces sp*. (CL190)	the inhibition of lipid peroxidation	α-tocopherol	The mycelial cake was stirred with acetone. The extract was concentrated in vacuo and extracted twice with ethyl acetate. The extract was dried and concentrated in vacuo. The fraction was applied to a silica gel column with n-hexane and ethyl acetate (4:1). The fraction was concentrated to dryness. The dry residue was rechromatographed on a silica gel column with chloroform, methanol, and ammonia (200:20:1). The elute was concentrated in vacuo and the residue was dissolved in chloroform and methanol (1:1) and purified by column chromatography on Sephadex LH-20 with the same mixture. The fraction was evaporated to dryness in vacuo and dissolved in ethyl acetate.	[319]
*Streptomyces* LK-3 (JF710608)	DPPH, MCA/ferrous ion, FRAP, β-carotene assay, NO^•^ scavenging activity	gallic acid	The crude extracts were diluted in water containing daidzein- 8-C-glucoside (puerarin), (−) gallocatechin gallate, sesamol, cyanidin-3-O-rutinoside, and delphinidinas.	[320]

Abbreviations: DPPH (2,2-diphenyl-1-picrylhydrazyl), CUPRAC (cupric reducing antioxidant capacity), FRAP (ferric reducing ability of plasma), TEAC (Trolox equivalent antioxidant capacity), EPR (electron paramagnetic resonance method), SNPAC (silver nanoparticles antioxidant capacity), BHT (butyl-hydroxytoluene), TE (Trolox equivalents), RP (reducing power assay), TPC (Total Phenolic Content), MCA (metal chelating activity assays), TBARS (Thiobarbituric Acid Reactive Substances), AAP (A. auricula-judae polysaccharide), EPS (Exopolysaccharide), SDM (A semi-defined medium), LPO (lipid peroxidation–hepatic lipid peroxidation), CAT (Antioxidant Enzymes Activity–catalase), GPx (glutathione peroxidase), TCA (trichloroacetic acid), ESR (electron spin resonance), XO (xanthine oxidase), GR (glutathione reductase), PMSF (phenylmethanesulfonylfluoride), CAA (cellular antioxidant activity), RT (room temperature), GSH (Reduced glutathione), CTAB (Cetyl Trimethyl Ammonium Bromide), CPC (Cetylpyridinium Chloride), CDM (Czapek Dox’s Medium), MEM (Malt Extract Medium), PDM (Potato Dextrose Medium), YEM (yeast extract glucose medium), YGA (yeast extract glucose agar), Astaxanthin (3,31-dihydroxy-β,β 1-carotene-4,41–dione), DMSO (dimethyl sulfoxide).

**Table 4:** Examples of biosynthesis of nanoparticles (NPs).

Shape/Size	Activity Assay/ Control	Biological Material	Effective Molecules	Preparation of Extract	Bio-Synthesis of NPs	Ref.
Silver (Ag) NPs, Absorbance at 430–450 nm						
spherical 410–450 nm	DPPH/ ascorbic acid	*Lantana camara* L.	terpenes	Powder (10 gm) of dried leaves was extracted with petroleum ether (30 mL) at RT for 6 h with shaking. It was treated with 30 mL of warm 10% aqueous KOH, shaken and two layers were separated. The petroleum ether layer was concentrated to dryness under reduced pressure.	One milliliter of concentrated extract was added to 6 mL of 1 mM AgNO_3_ at RT, and kept in the dark for 24 h. The slurry was dried under vacuum.	[392]
spherical 5–38 nm	DPPH/ ascorbic acid	*Costus afer*	Carbohydrates flavonoids, phenolics, alkaloids, organic acids	Fresh leaves were air-dried. Two grams of the powder was macerated with 150 mL DW and heated at 90 °C for 1 h.	Eighty milliliters of filtered extract was mixed with 400 mL of 1 mM AgNO_3_. The mixture was stirred at 90 °C for 120 min.	[393]
spherical 5–30 nm	ABTS, DPPH, NO f.r.s.a.	*Taraxacum* *officinale*	flavonoids, primary aromatic amines, terpenoids, triterpenes	The dried leaves were powdered and sieved. Five grams of powder was added to 50 mL of DW and boiled at 60 °C for 15 min, followed by cooling and filtration.	The extract was mixed with AgNO_3_ (1 mM) with a 1:5 ratio for 15 min. at pH 6.0, at RT.	[394]
spherical 12–40 nm.	DPPH, ABTS, O_2_^●−^, NO^•^ f.r.s.a.	*Morus alba*	carbohydratesproteins, secondary metabolites	Ten grams of the chopped leaves were refluxed with 100 mL DDW for 60 min. The product was filtered and centrifuged at 2000 rpm for 5 min.	Ten milliliters of extract was mixed with 90 mL AgNO_3_ solution with stirring for 10 min.	[395]
spherical 50–60 nm	DPPH/BHT	*Thymus kotschyanus*	phenolic, flavonoid compounds	The plant was washed, dried at 25 °C, powdered with mortar. Two grams of powder was added to 300 mL of boiling water and kept for 30 min. The obtained extract was filtered.	The extract (10 mL) was mixed with 100 mL of 1 m M aqueous solution of AgNO_3_ at RT and stirred for 30 min in a dark place.	[396]
spherical 5–45 nm	DPPH, H_2_O_2_, OH^•^, O_2_^●−^ f.r.s.a.	*Cestrum nocturnum*	phenolic compounds, amines, amides, aldehydes, nitriles, flavonoids, tannins	The leaves were dried and powdered. Eight grams of powder was added to 100 mL DI and heated at 70 °C for 2 h. The extract was centrifuged at 3000 rpm for 5 min followed the filtration.	Twenty milliliters extract was stirred with 180 mL 1 mM AgNO_3_ solution for 5 min at RT.	[397]
spherical 5–50 nm	DPPH, FRAP, TAC/ascorbic acid	*Streptomyces naganishii (MA7)*	Proteins, enzymes	The strain was inoculated into 50 mL of ISP 2. The mycelium was centrifugated at 5000 rpm for 30 min.	Five grams of wet biomass was exposed to 50 mL of 1 mM AgNO_3_. The mixture was incubated for 28 °C at 120 rpm, and then ultracentrifugation.	[398]
variables 150–250 nm	DPPH, FRAP, TAC	*Parmeliopsis ambigua*, *Punctelia subrudecta* *Evernia mesomorpha*, *Xanthoparmelia plitti mycelia* *mats*	polyphenols, native proteins	The cultures were inoculated on MYE. The plates were incubated at 28 °C. After 7–10 days, the isolated mycobiont was subcultured into a fresh medium. The mycobiont was grown aerobically in MYE at 28 °C with shaking at 150 rpm. After 10 days, mycelia were separated by filtration.	The mycelia mats were mixed separately with 100 mL SDW and 1 mM AgNO_3,_ and incubated at RT on a rotary shaker at 150 rpm. The reaction was carried out in bright conditions for 24 h.	[399]
spherical 15–30 nm	DPPH	marine algae *Ecklonia cava*	polyphenols, polysaccharides, amine, amide species	Five grams of powder and 500 mL of DW were kept at 100 °C for 1 h. Then, the mixture was centrifuged at 3000 rpm for 20 min, and filtered by a filter paper.	Ten milliliters of aqueous extract was mixed with 90 mL of 1 mM AgNO_3_ solution and stirred for 72 h. AgNPs were lyophilized.	[400]
spherical 2–10 nm	DPPH, H_2_O_2_ f.r.s.a.	*Pestalotiopsis microspora* VJ1/VS1	phenolic compounds, proteins	The culture was cultivated in 100 mL of PDB at 25 °C. After 6 days fungal biomass was transferred to 100 mL of SDDW, boiled, and filtered.	10 mL of filtrate was incubated with 90 mL of 1 mM AgNO_3_ in darkness for 24 h at RT.	[401]
100 nm	DPPH/ ascorbic acid	*Cladosporium cladosporioides* -	NADPH-dependent reductase, phenolic compounds, proteins	The mycelial was grown in PDB for 72 h. The biomass was filtered and then incubated at RT for 48 h in 100 mL DW.	Ten milliliters of filtrate was added to 90 mL of 1 mM AgNO_3_.	[402]
spherical 3–40 nm	DPPH/ ascorbic acid	*Aspergillus versicolor* ENT7-isolated from the ethnomedicinal plant *Centella asiatica*.	-	The fungal isolate was grown in 100 mL of PDB at 26 °C with shaking at a speed of 100 rpm. After the seventh day, the fungal biomass was separated and washed with SDDW. 10 g of biomass was mixed with 100 mL SDDW and kept at 28 °C for 72 h in a constant shaking.	The aqueous solution was filtered (100 mL) and added to 100 mL of 1 mM of silver nitrate and incubated at 28 °C for 24 h in dark condition.	[403]
15–25 nm	DPPH	*Trichoderma atroviride* KNUP001	-	The freshly prepared mycelial filtrate was prepared by aerobically growing in PDB with the agitation of 180 rpm at 28 °C for 4 days. Then, the biomass was filtrated and washed SDDW. The biomass (20 g) was ground in 100 mL of deionized water and filtered.	The filtrate (100 mL) was mixed with AgNO_3_ (5 mM or 10 mM) and the solution was kept at 40 °C under darkness.	[404]
spherical 65 nm	DPPH, FRAP/ascorbic acid	endophytic fungi, Penicillium species of Glycosmis mauritiana	tannins, saponins, terpenoids flavonoids,	Sterilized (HgCl_2_, 1 mg ml^−1^) bark material was incubated in PDA at RT for 7–8 days. The isolated fungi were cultured in PDB for 10 days. The mycelial mat was centrifuged (6000 rpm, 10 min) and the supernatant was shaken for 24 h.	Eighty milliliters of 3 mM AgNO_3_ was added to 20 mL of extract. NPs were centrifugated at 7000 rpm for 10 min.	[405]
spherical 15–35 nm	ABTS/BHT	*Inonotus obliquus*	proteins	Ten grams of mushrooms were washed, crushed mixed with 200 mL DDW, and stirred for about half an hour.	Five milliliters of the filtered solution was mixed with 95 mL of 1 mM AgNO_3_ at RT for 80 min.	[406]
spherical	FRAP, DPPH, /ascorbic acid	*Cladosporium*	carbohydratestannin, phenolic glycosides, terpenoids, alkaloids, phenol anthraquinones, flavanones	The species was cultured using PDB for 15 days at RT.	Five grams of dried and milled mycelia mat was mixed with 20 mL of SDDW, The mixture was heated to 100 °C for 10 min. Then, 10 mL of 5 mM AgNO_3_ was added.	[407]
10–80 nm	DPPH	*Agaricus bisporus*, *Ganoderma lucidum*	flavoproteins, lysine, tryptophan, glutamic acid, riboflavin	Fresh mushrooms were washed with DDW, dried for 4 days, powdered. 1 g of powder was added to100 mL of DDW, and stirred for 60 min.	The filtered extract (10 mL) was added to 90 mL of 1 mM AgNO_3_. This solution was kept at RT for 12 h or heated at 60 °C for 5 h.	[408]
spherical 15–22 nm	DPPH/trolox, ascorbic acid	*Ganoderma lucidum*	proteins, steroids, nucleotides, amino acids, terpenoids, phenols, vitamins, glycoproteins, poly-saccharides	Five grams of powdered mushrooms were added to 100 mL of 70% ethanol solution. The extract was prepared by the microwave-assisted process.	Twenty milliliters of filtered extract was diluted to 100 mL by DDW, and then 15 mg of AgNO_3_ was added and mixed by the magnetic stirrer system.	[409]
spherical 10–30 nm	DPPH	*Ganoderma lucidum*	polyphenol, carbonyl species, amino acid	The sample was washed with DW and dried at 40 °C for 3 days. The dried sample was grounded into a powder. 5 g of powder was extracted using water (20 mL via Soxhlet extractor at 80 °C for 8 h. The extract was filtered, and concentrated to 100 mL under 60 °C in a rotary evaporator.	Ten millilitres of extract was added to 90 mL of 1 mM AgNO_3_ solution and incubated at 60 °C in dark, with an interval the stirring for 4 h of incubation. Ag-NPs were collected by centrifugation at 10,000 rpm for 30 min at 4 °C. The pellet was washed and dried at 60 °C.	[410]
spherical 5–20 nm	DPPH/ ascorbic acid	*Streptomyces griseorubens* AU2	-	The pure culture was inoculated on ISP-2 broth and incubated at 28 °C and 130 rpm for 7 days. After that, the culture was centrifuged at 4000 rpm for 20 min and the biomass was washed with DW, suspended in DW, and incubated at 28 °C and 130 rpm for 48 h, and finally centrifuged at 4000 rpm.	Ten milliliters of supernatant with 50 mL of 1 mM AgNO_3_ were incubated at 28 °C and 130 rpm for 48 h.	[411]
spherical 12–16 nm	DPPH, ABTS, FRAP	*Raphanus sativus* L.	-	The fresh leaves were washed, pat dried, and chopped, shade-dried to constant mass at RT. Ratio: product/solvent was kept at 1:12 *w*:*v*, extraction time: 3 h. mechanical stirring, temperature: 70 °C (hydroalcoholic mixture), 67 °C (ethanol); microwave-assisted extraction: time:10 min. at 140 °C, max. power 1000 W.	One hundred milliliters of each filtered extract were mixed with 100 mL of 10 mM aqueous AgNO_3_ solution and incubated at RT for 30 min.	[412]
Gold (Au) NPs, Absorbance at 530–535 nm						
multiply twinned quasi- spherical 5–35 nm	DPPH	*Acroscyphus* *sphaerophoroides Lev*, *Sticta nylanderiana*	carboxylic acids, esters, phenols, quinones	The samples were cleaned with DDW, shade dried, and ground in a glass mortar.	One gram of powder was stirred with 100 mL aqueous solution (10^−3^ M) of HAuCl_4_, at RT for 12 h. The supernatant was centrifugated (10,000 rpm). The biomass was washed with DDW and dried.	[413]
Spherical 5–15 nm	DPPH	*Lemanea fluviatilis* (L.)	proteins	The red alga samples were cleaned by DW and then dried for a one week in a dark place.	One gram of powder was stirred with 100 mL aqueous solution (10^−3^ M) of HAuCl_4_, at RT for 12 h. The supernatant was centrifugated (10,000 rpm). The biomass was washed with DDW and dried.	[143]
spherical 79 nm	-	*Tetraselmis suecica*	water-soluble heterocyclic compounds	The cultures were harvested on the sixth day, then centrifuged at 2000 *g* for 10 min at 4 °C. The biomass was washed with 0.9% NaCl and centrifuged. The cells were damaged in a mortar in a presence of liquid N_2_ and then again centrifuged under the same conditions.	The cell extracts were added to 4 mL of 1 mM HAuCl_4_. The mixtures were incubated in a water bath. The recommended conditions: 1 mL of extract, 5 min of incubation, 90 °C of incubation temperature.	[414]
triangular, circular, hexagonal	DPPH/ ascorbic acid	*Escherichia coli*	-	*E. coli* was grown in a nutrient broth at 25 °C under agitation at 180 rpm. The biomass sieved and washed with DW. 1 mg of biomass mixed with 50 mL of SDW, and after 24 h, precipitated by NH_4_(SO_4_)_2_. The pellet was dissolved in phosphate buffer (0.05 M, pH 8.0) and dialyzed.	Five milliliters of solution (50 mg of HAuCl_4_ in 250 mL of water) was mixed with 24 or 30 mL of the protein solution and vigorously stirred for 4 h.	[415]
spherical	DPPH, OH, O_2_^●−^, NO^•^ f.r.s.a.	*Solanum torvum*	-	The dried fruit was made to a fine powder. The 1% of aqueous extract was obtained by using soxhlet apparatus.	Eight milliliters of extract was mixed with 2 mL of 1 mM HAuCl_4_ and incubated at RT for 24 h, then the mixture was centrifuged at 10,000 rpm for 10 min. The pellet was re-suspended in ethanol.	[416]
spherical 13–15 nm	-	*Phormidium valderianum*, *P.tenue*, *Microcoleus chthonoplastes*, *Rhizoclonium fontinale*, *Ulva intestinalis*, *Chara zeylanica*, *Pithophora oedogoniana*	-	The samples were cultured in an artificial seawater medium. Algal biomass was mixed with betadine and antibiotic mixtures. After 12 the biomass was washed with SDW.	Au-loaded biomass was obtained by its expose to 15 ppm Au (III) solution at pH (5, 7, 9). After 72 h, it was washed with SDDW, and dried on air. The biomass was sonicated for 30 min with 7.5 mM sodium citrate, followed by centrifugation of 5 min at 3000 rpm.	[417]
100 nm	DPPH, FRAP	Cladosporium cladosporioides	NADPH- dependent reductase, phenolic compounds	The endophytic fungal isolates were cultured using PDB for 21 days at 25 to 28 °C. The biomass was filtered and washed with DW. This biomass was incubated at RT for 48 h in 100 mL DW.	A 1 mM HAuCl_4_ solution was mixed with the fungal suspension filtrate.	[402]
spherical, triangle, hexagonal rod 23 nm	ABTS	*Inonotus obliquus*	proteins	Ten grams of cut mushrooms were stirred with 100 mL of DDW, for 30 min. Then, the solution was filtered through Whatman filter paper.	The extract (5 mL) was added to 95 mL, 1.0 mM HAuCl_4_. The mixture was stirred at RT for 30 min.	[418]
spherical 5–30 nm	DPPH	*Lactobacillus kimchicus* DCY51T 19	-	Bacterial cells isolated from kimchi were inoculated into 100 mL MRS broth and incubated at 37 °C for 24 h. After incubation, the broth was centrifuged at 6300× *g* for 5 min.	The biomass was washed with SDW and resuspended in 15 mL of SDW. Then, 1 mM of gold salt was added. The mixture was incubated at 30 °C and shaken at 150× *g* in darkness. The product was centrifuged at 2500× *g* for 5 min.	[419]
spherical 8–50 nm	DPPH	*Enterococcus* species	proteins and other nitrogenous molecules	A distinct colony of each strain was used to inoculate 10 mL of sterile broth and incubated at 37 °C for 18 h. Then, the cultures were centrifuged at 4000 rpm at 10 °C for 15 min.	One milliliter of the cell-free extract and 30 mL of 1 mM HAuCl_4_ solution were mixed.	[420]
8–12 nm	-	*Sargassum wightii*	-	Seaweed was cleaned, dried for 3–5 days, ground to powder.	One gram of seaweed powder was added to 100 mL of 1 mM HAuCl_4_ solution within 12 h in a stirring condition.	[421]
Spherical, cubic 15–60 nm	-	*S. platensis*	-	The strain was cultivated in a standard Zaroukh water-salt nutrient medium. After 5–6 days of cultivation, the bacterial cells were harvested and then were washed in DW.	The wet biomass (1 g) was mixed with 100 mL of HAuCl_4_ solution (10^−2^–10^−4^ M). The mixture was shaken for 5 days at RT.	[422]
20–70 nm	DPPH, NO^•^ f.r.s.a.	*Vitex negundo*	Flavonoids, polyphenols	Leaves were dried for 3 days in a dark place. The biomass (10 g) was stirred with DDW (50 mL) for 12 h at 500 rpm. The extracts were filtered and lyophilized.	Lyophilized extract (0.5 g) was reconstituted in 5 mL DDW at 100 µgmL^−1^. To 1 mL of extract, 20 mL of HAuCl_4_ (0.01 M) was added drop-wise and stirred at 500 rpm. The solution was kept overnight.	[423]
Zinc oxide (ZnO) NPs, Absorbance at 340–360 nm.						
hexagonal 10–61 nm	DPPH	*Pichia kudriavzevii*	amino acids	The yeast was grown on PDB in a vibrating incubator at 150 rpm for 72 h at 28 °C. Mycelia were centrifugated (10,000 rpm, 10 min, 4 °C), washed with SDW. 20 g of biomass was suspended in 100 mL of SDW and incubated for 72 h. Then, biomass was filtrated.	One hundred milliliters of filtrate was added to10 mL of 10 mM Zn(Ac)_2_·2H_2_O, incubated at 35 °C with agitation at 150 rpm for 12–36 h. The biomass was centrifugated at 10,000 rpm for 10 min and dried at 150 °C for 6 h.	[424]
20–40 nm	DPPH	*Berberis aristata*	polyphenols, alcohol, carboxylic acid, ether ester amino acid	The leaves were washed, dry at RT. Later 10 g of leaves were cut, soaked in 100 mL of DDW, heated at 50 °C for 10 min., and filtered.	Sixty milliliters of extract was heated to 70 °C and stirred with 0.1 M Zn(Ac)_2_·2H_2_O) at basic conditions. Then, the solution was centrifuged at 6000 rpm for 20–25 min.	[425]
Selenium (Se) NPs, Absorbance at 510 nm						
10–250 nm	DPPH, FRAP, TAC	*Streptomyces minutiscleroticus* (M10A62)	protein, peptide, amine, amide compounds	A 0.1 g soil sample was plated in starch casein agar plates enriched with nystatin (100 µg/mL) and nalidixic acid (20 µg/mL). The strain was transferred to 100 mL of MYE broth and incubated in a rotator shaker (200 rpm) for 5 days, and centrifugated at 5000 rpm for 30 min	Five grams of biomass washed with SDDW was mixed with 100 mL of an aqueous solution of 1 mM Na_2_SeO_3_ and kept in a rotator shaker for 72 h.	[426]
30–300 nm	DCF in HUVEC	*Pantoea agglomerans* strain UC-32	-	Bacterial cells were cultivated in TSB enriched with 1 mM Na_2_SeO_3_ at 25 °C.	Cell suspensions were sonicated at 100 W for 2 min and centrifuged at 10,000× *g* for 10 min. Pellets were suspended in SDS 0.1%/1 M NaOH, and centrifuged.	[427]
spherical tetragonal 14–26 nm.	DPPH/ ascorbic acid	*Ephedra aphylla*	phenolic compounds, flavonoid tannin	Twenty grams of the dried plants were shaken with 200 mL DW for 30 min in a water bath at 70 °C. The mixture was filtered.	Twenty milliliters of 1 mM selenium sulfate was stirred with 20 mL of the plant extract for 2 h at RT.	[428]
Copper (Cu) NPs Absorbance at 350–380 nm.						
spherical 60–90 nm	DPPH/ ascorbic acid	*Cissus arnotiana*	-	One gram of the powder of the dried leaves was added to 100 mL DDW, boiled at 70 °C for 30 min. The mixture was filtered.	Ten milliliters of the extract was stirred with 90 mL of 10 mM of CuSO_4_, for 4 h at RT. The mixture was centrifugated at 10,000 rpm for 5 min., The pellet was washed with DDW, and ethanol.	[382]
12–16 nm	DPPH, NO^•^, O_2_^●−^ f.r.s.a.	*Dioscorea bulbifera*	ascorbic acid	The washed and sliced tubers were dried in a dark place for 3 days. Five grams of the obtained powder and 100 mL of SDW were boiled for 5 min. The extract was filtered	Five milliliters of extract was shaken at 150 rpm in the dark place at 40 °C with 95 mL of 1 mM CuSO_4_·5H_2_O.	[429]
Copper oxide (CuO) NPs Absorbance at 280–360 nm						
1.5–20 nm	-	*Lens culinaris*	primary and secondary amines, aldehydes, phenols, proteins	The plant was homogenized in mortar. After that, 100 mL of distilled water was added.	1 mM CuSO_4_ was stirred with the filtrated extract (ratio: 1:5, *v*/*v*) for 1 h at 37 °C (pH 9). Then, it was centrifuged at 12,000× *g* for 15 min. The pellet was washed with DW, re-suspended in DW and ultra-sonicated.	[430]
10 nm	DPPH	*Galeopsidis herba*	flavonoids, phenolic acids,poly- saccharides	A total of 4.5 g powdered plants were mixed with 300 mL DDW, and stirred for 50 min at 85 °C. Then, the mixture was filtered.	The extract was mixed with Cu(NO_3_)_2_ in the proportion: 90:1 (*w*/*w*), and vigorously stirred for 4 h at 80 °C.	[431]
Spherical, agglomerated	-	*Terminalia phanerophlebia*	-	Extract from the oven-dried leaves was prepared from 2 g of the ground powdered and 150 mL deionized water, ethanol, or acetone. The extracts were filtered.	Thirty milliliters of CuSO_4_·5H_2_O (0.1 M) was stirred with 10 mL of the plant extract, and heated at 90 °C for 5 h. The solution was kept overnight at RT. The CuO NPs were centrifuged, washed with DW, dried in hot air.	[432]
Iron (Fe) NPs Absorbance at 214 nm						
Spherical, cubic 43–220 nm	DPPH	*Amaranthus dubius*	amaranthine, phenolic compounds	The leaves were cleaned, chopped into small pieces. 20 g of leaves were mixed with 100 mL DW and keep at 50 °C for 45 min. The mixture was filtered.	The leaf extract (pH 6) was added a drop to 0.5 M FeCl_3_ with stirring for 90 min.	[433]
20–25 nm	DPPH, ABTS, H_2_O_2_ f.r.s.a.	*Asphodelus aestivus* Brot.	phenolic compounds, poly- sachharides	The infusion was prepared in a ratio of 5%. The filtrate was concentrated using a vacuum evaporator.	Five milliliters of extract was mixed with 5 mL of 1 mM aqueous FeCl_3_. The mixture was kept at 50–60 °C for 20 min with shaking. Then, it was centrifuged at 5000 rpm for 30 min.	[434]
Iron oxide (FeO) NPs Absorbance at 290 nm						
spherical 58–530 nm	DPPH	*Amaranthus spinosus* L.	amaranthine, compounds with hydroxyl or amines groups, free amino, carboxylic moieties	Ten grams of fresh leaves were washed with DW, and chopped into pieces, and mixed with 50 mL water, and keep at 50 °C for 45 min. The supernatant was filtered.	The leaf extract (pH 6) was added to 50 mL of 0.5 M FeCl_3_ stirring at 37 ± 1 °C for 90 min. The precipitate (FeO NPs) was washed with ethanol and dried at 60 °C for 180 min.	[383]
Nickel oxide (NiO) NPs Absorbance at 305 nm						
spherical agglomerated NPs 20–50 nm	DPPH	stevia leaf broth	terpenoids, polyphenols, proteins, aldoses	To 5 g of dried leaves, 100 mL DW was added and boiled (2 min.), and finally filtered.	One gram of nickel acetate in 200 mL DW was stirred with 25 mL extract for 2 h. The mixture was then heated at 100 °C, and then at 500 °C for 2 h.	[435]
agglomerated NPs	TAC/phosphomolybdenum, DPPH	*Berberis balochistanica*	polyphenols, carboxylic acids, alcohols, sulfur compounds	The material was washed, oven-dried for 10 h at 40 °C. 20.66 g of powder was stirred with 200 mL of DW for 12 h. Then, the extract was filtered and centrifuged at 3000 rpm for 30 min.	A 50 mL extract was added drop by drop to the solution of NiNO_3_ (0.3 M). The mixture was heated at 60 °C with stirred at 500 rpm for 3 h.	[436]
Manganese (Mn) NPs Absorbance at 415–417 nm						
spherical granular 57–69 nm	-	*Ctenolepis garcini* (Burm. f.)	native proteins	To 2 g air-dried sample, 30 mL of SDW was added, and boiled (2 min.).	Five milliliters of the filtered extract was added to 25 mL of 1 mM KMnO_4_ solution and stored in RT for 24 h.	[437]
Manganese oxide (MnO) NPs Absorbance at 460 nm						
spherical 80± 0.5 nm	-	*Abutilon indicum*	-	Twenty grams of leaves powder was mixed with 50% methanol. It was placed on a magnetic hot plate and underwent stirring for about 30 min at 55 °C and allowed to settle overnight.	One hundred milliliters of 0.1 M MnSO_4_·H_2_O was mixed with 100 mL of plant extract. A total of 0.1 M NaOH solution was added dropwise to the beaker with constant stirring for about 1 h at pH 8.0 and 50 °C.	[438]
Magnesium Oxide Nanoparticles (MgO) NPs Absorbance at 250 nm						
Spherical 7–40 nm	-	*Penicillium chrysogenum*	polysaccharides hydrocarbons, amines, carboxylate, amino groups	The fungal strains were inoculated into MAB, and incubated for 5 days at 30 ± 2 °C and shaking state at 150 rpm. Then, the biomass was centrifuged and resuspended in 100 mL in DDW.	A total of 76.9 mg of Mg(NO_3_)_2_.6H_2_O was dissolved in 10 mL DW, mixed with 90 mL of biomass filtrate and incubated for 24 h. The white precipitate was collected and rinsed with DW, and oven-dried at 400 °C for 3 h.	[439]

Abbreviations: room temperature (RT); double-distilled water (DDW), tryptic soy broth (TSB), human umbilical vein endothelial cells (HUVEC), starch casein agar medium (SCA), malt extract broth media (MAB), Malt Yeast Extract medium (MYE), sterile double distilled water (SDDW), double distilled water (DDW), sterile distilled water (DW), deionized water (DI), potato dextrose broth (PDB), potato dextrose agar (PDA), International Streptomyces (ISP 2), dichlorofluorescein (DCF), free radical scavenging activity (f.r.s.a).
